# Chasing Bubbles: Toward a Standardized Approach for Quantifying Methane Ebullition Using Bubble Traps in Streams and Rivers

**DOI:** 10.1021/acs.est.6c13075

**Published:** 2026-10-23

**Authors:** Adam Bednařík, Eva Darenova, Helena Cvetkovic, Charlotte Doebke, Clemens Karwautz, Lukáš Kokrda, Natalia Kowalska, Katrin Attermeyer

**Affiliations:** † Department of Matters and Energy Fluxes, 117207 Global Change Research Institute of the Czech Academy of Sciences, Belidla 4a, 603 00 Brno, Czech Republic; ‡ Department of Functional and Evolutionary Ecology, 27258 University of Vienna, Djerassiplatz 1, 1030 Vienna, Austria; § WasserCluster Lunz- Biologische Station, Dr. Carl Kupelwieser Promenade 5, 3293 Lunz am See, Austria

**Keywords:** greenhouse gas, methane emissions, bubble trap, chamber, stable carbon isotopes

## Abstract

Ebullition transports large quantities of the potent greenhouse gas methane (CH_4_) from stream sediments into the atmosphere. However, the use of different sampling designs complicates comparisons of CH_4_ emissions estimates between studies. Here, we present the first systematic review of sampling designs used in the past for assessing fluvial CH_4_ ebullition rates. In addition, we conducted field experiments to estimate the optimal number of bubble traps and the length of the sampling period, and to assess uncertainties in determining CH_4_ content in the emitted bubbles. Our results demonstrate considerable spatial variability in bubble fluxes (0–4.8 L m^–2^ d^–1^) and CH_4_ content in the bubbles (0.01–67%), necessitating a larger number of bubble traps than typically deployed in streams. Using fewer traps leads to greater deviations in CH_4_ ebullition estimates than shorter deployment times. Therefore, the robust sampling design should account for strong small-scale heterogeneity in streams through an adequate number of bubble traps and link bubble fluxes to trap-specific gas content. It should also consider the risk of CH_4_ concentration decreases in accumulated bubbles over a few days, primarily due to redissolution, followed by oxidation and leakage.

## Graphical abstract

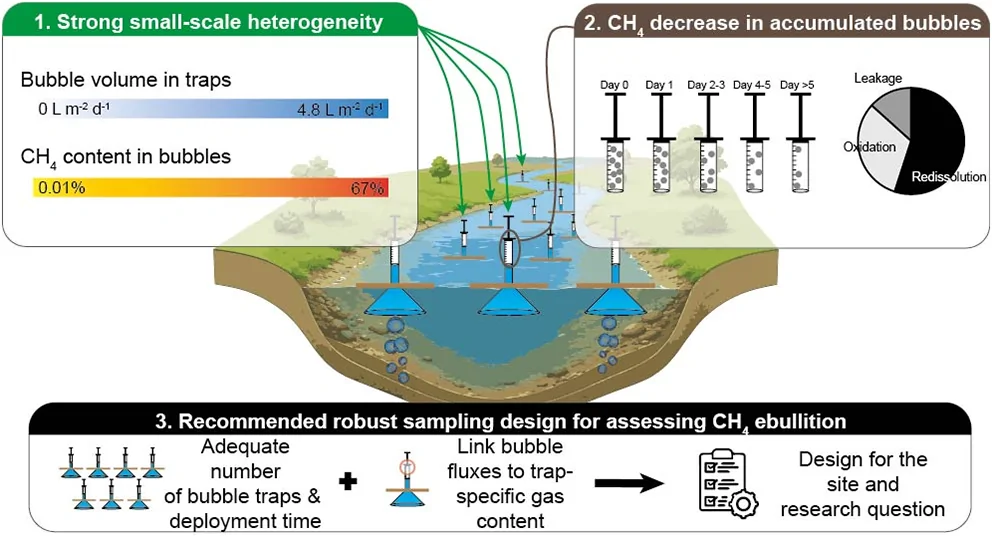

## Introduction

Streams and rivers have been recognized to emit methane (CH_4_) to a similar extent as lakes or reservoirs, but a limited number of CH_4_ ebullition measurements still hampers more robust estimates of CH_4_ fluxes from running waters.^1,2^ Recent estimates show that diffusive and ebullitive fluxes are similar in magnitude in rivers.^2^ However, the CH_4_ flux assessments from streams and rivers are dominated by measurements of diffusive fluxes, with ebullition measurements accounting for only 8% of the records.^3^ These studies report a high spatiotemporal variability of ebullition in streams, given by a wide range of both bubble fluxes and bubble CH_4_ contents.^4,5^ Thus, there is a critical need for a robust and standardized sampling design for stream ebullition assessments.

There are three main approaches for measuring CH_4_ ebullition—floating chambers, bubble traps, and hydroacoustic surveys. When the chambers are connected to a portable analyzer or equipped with a CH_4_ sensor,^6,7^ ebullition is estimated based on abrupt increases in the CH_4_ concentration in the chamber. Alternatively, the calculated diffusive fluxes using Fick’s law are subtracted from the total fluxes (diffusive + ebullitive) measured by the chambers in the case of manual gas sampling.^8^ Chamber measurements do not provide information on bubble volume and associated CH_4_ content and are typically conducted over minutes to hours. In the case of bubble traps, often using an inverted funnel design, the combination of the volume of the captured bubbles and their CH_4_ content is used to estimate CH_4_ emission through ebullition. Bubble volumes and subsequent gas analyses are performed manually, or automatically if traps are equipped with pressure and gas sensors, allowing determination of the volume and CH_4_ content of accumulated bubbles.^9,10^ Automated bubble traps can provide highly detailed time series of bubble fluxes, enabling temporal variability and its drivers to be identified.^11,12^ However, the automated bubble traps are mainly used in reservoirs because their use may be limited in shallow streams due to their larger size. The number of traps deployed varies in different studies, as well as the measurement periods, which range from hours to months.^5,13^ In addition, bubble traps vary in design, material used, and size, as well as in their distribution across the site. There is also no consensus on the sampling design or uncertainties related to the chosen sampling strategy. The only existing estimate of the optimum number of traps and sampling period for streams was made by Robison et al.,^14^ who recommended deploying 10 traps over 60 days to cover the spatiotemporal variability of CH_4_ ebullition. A similar estimate of the optimal number of traps (11) deployed over 39 days was proposed using depth-stratified sampling also for lakes.^15^ A partial comparison of the use of floating chambers and bubble traps for estimating ebullition in rivers shows that both methods can produce consistent results.^16^ However, ebullition may be underestimated if floating chambers are deployed for only tens of minutes.^17^ Finally, hydroacoustic surveys use echo sounders to detect rising bubbles and determine their volume and density over large areas in a short time.^18,19^ This method is widely applied to quantify CH_4_ ebullition in reservoirs and lakes but has limited applicability in shallow, fast-flowing rivers due to depth-related constraints, and is therefore primarily used in deeper river sections, such as upstream of dams.^20^ To date, no systematic overview of the designs used to assess fluvial CH_4_ ebullition has been reported.

Open questions remain, particularly regarding the correct use of the bubble traps. To accurately determine ebullitive fluxes using bubble traps, the volumes of gas collected in bubble traps must be coupled with a specific CH_4_ content to estimate ebullition. Basically, there are two ways to sample the gas for analysis of its content: either by sampling the accumulated gas from the traps^17^ or by collecting fresh bubbles after disturbance of the sediments near the traps.^21^ Studies from various ecosystems suggest that the concentration of trapped CH_4_ may decline rapidly, potentially due to oxidation or dissolution.^9,22^ This complicates the accurate estimation of CH_4_ concentrations in the released bubbles. Similarly, sampling of sediment-disturbed bubbles raises concerns about their comparability to naturally released bubbles, which have rarely been assessed. Thus, although Wik et al.^15^ and Robison et al.^14^ evaluated the number of traps and the deployment time in lakes and streams, respectively, further questions remain regarding the data supporting the best gas sampling practices used to determine the CH_4_ content for CH_4_ ebullition estimates. Consequently, the comparability of ebullition estimates between studies is challenged by different sampling designs used.

The aims of this study were (1) to present a systematic review of the sampling designs used in the past for assessing fluvial CH_4_ ebullition rates and (2) to provide a recommendation for a robust sampling design for bubble trap assessments in streams based on experimental measurements. Specifically, we assessed the optimal number of bubble traps and the length of the sampling period in streams, including the optimal method of gas sampling for the determination of CH_4_ content. We increased the number of bubble traps and shortened the sampling period compared with previous studies to limit the effect of temperature changes on the temporal variability of CH_4_ ebullition. Additionally, we focused on a detailed observation of the CH_4_ concentration changes in trapped bubbles over time.

## Methods

### Systematic Review of the Sampling Designs

Relevant studies were identified through searches in the Web of Science and Scopus databases as well as from published data sets on fluvial CH_4_ fluxes.^3^ The assessment of methodology and reporting was conducted based on predefined inclusion and exclusion following standardized criteria according to the “Preferred Reporting Items for Systematic Reviews and Meta-Analyses” (PRISMA) method.^23^ Briefly, we searched English-language literature for studies reporting field measurements of CH_4_ emissions, including ebullition, in various types of running waters. A comprehensive search of Web of Science and Scopus on 29 November 2024 identified 3,659 studies, from which 42 unique studies were included in the final data set based on the selection criteria (Figure 1). Details of the search strategy, including keywords, inclusion criteria, and results, are described in the Supporting Information: Details of systematic review of the sampling designs.

**Figure 1:**
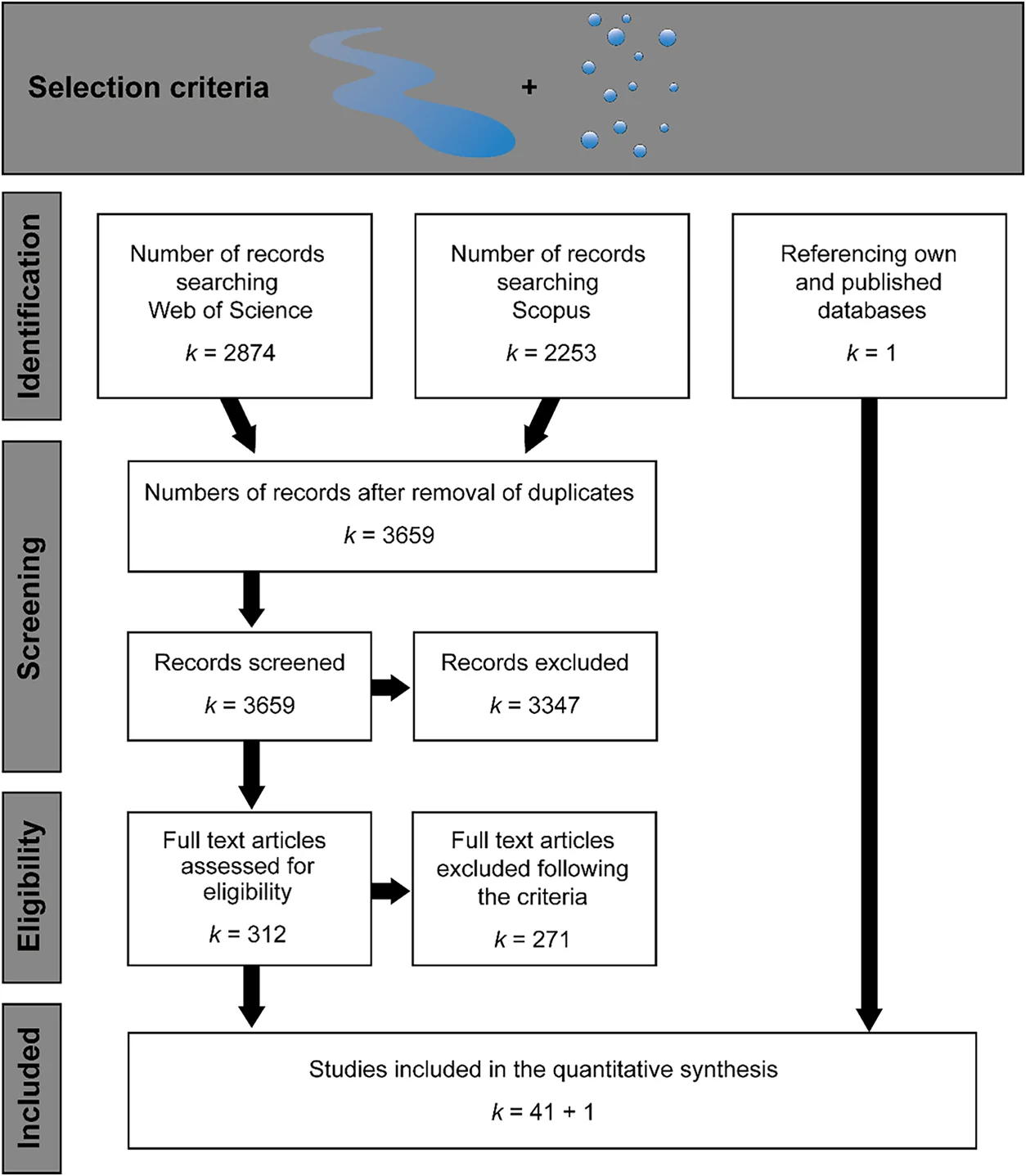
PRISMA diagram showing the results of the search strategy for studies assessing fluvial CH_4_ ebullition. Forty-one unique studies were included in the data set based on the selection criteria, and one additional study that did not reference running waters in its title or abstract was added based on comparisons with the Global River Methane Database of concentrations and fluxes by Stanley et al.^3^ and our own collection.

### Assessment of Robust Sampling Design for Stream Ebullitive Fluxes

To generate recommendations for bubble rate measurements, we tested the optimal number of bubble traps and the length of the sampling period in Jihlava and Hana streams in Czechia, and in Schweinsbach stream in Austria (1). To generate recommendations for bubble CH_4_ content measurements, we monitored the concentration of CH_4_ and δ^13^C-CH_4_ in trapped bubbles over time in the streams Hana and Kyjovka in Czechia (2.1) and performed additional experiments and calculations to explain the observed changes. We tested for syringe leakage to determine whether the material and connections were permeable to CH_4_ (2.2), and assessed the potential effects of CH_4_ oxidation (2.3) and bubble redissolution (2.4). Finally, we compared the CH_4_ concentration and δ^13^C-CH_4_ in bubbles naturally and artificially released from stream sediments in Kyjovka (3). Characteristics of the investigated streams are summarized in Table 1.

(1) We deployed 20 bubble traps in stream sections of about 100 m in length at three streams (60 traps in total) for 30 days. Bubble traps consisted of an inverted plastic funnel (33.8 cm diameter, surface area 0.09 m^2^), floating material (styrofoam) preventing traps from tilting in stream current, and a 60 mL plastic luer-lock syringe with a three-way stopcock attached to the top of each funnel to allow sampling of the trapped gas (Figure S2). The traps were held in position by a string attached to stainless steel rods hammered into the sediments. Traps were systematically deployed in the stream sections to cover a range of water depths (0.1–0.9 m) and velocities (0–0.5 m s^–1^). The volume of accumulated gas was recorded every 3–4 days, and at least two samples were taken from each trap for later analysis of CH_4_ concentrations of the trapped bubbles. Details on CH_4_ concentration analysis are given in the Supporting Information: Analysis of gas samples. We performed simulations of hypothetical sampling scenarios to determine the optimal number of sampling days and number of bubble traps based on the generated data sets structured into bins. All possible combinations of daily or trap ebullition averages were considered. Data analysis followed the principles given by Wik et al.,^15^ and details are given below.

(2.1) To assess the changes in concentration of CH_4_ and δ^13^C-CH_4_ in trapped bubbles over time, two bubble traps were placed in each of the two streams in a box with a full bottom (Figure S3) immersed in the streamwater, where water exchanged through two holes (50 cm^2^) in the box to mimic in situ conditions. We injected two different volumes of gas (50 and 150 mL) containing ∼60% CH_4_ into the traps. Immediately after injections, we sampled gas for later analysis of CH_4_ concentration and δ^13^C-CH_4_ by withdrawing 0.1 mL of gas with a gastight syringe and injecting it into a 12 mL vial (Labco, U.K.) that had been previously flushed with synthetic air without hydrocarbons (Messer Technogas, CZ). The traps were repeatedly sampled over a period of 13 to 18 days. The streamwater temperature at the monitoring sites ranged from 18 to 23 °C during the sampling periods. We selected two different volumes of gas in order to evaluate how the rate of change in concentration depends on its volume. The volumes used correspond well to the bubble volumes recorded during our experiment on estimating the optimal number of traps, in which the volumes after a few days were mostly in the range of a few tens to a few hundred milliliters.
(2.2) We verified the extent of syringe leakage by storing a gas mixture (natural bubble from sediment with ∼50% CH_4_) in a syringe (130 mL) at room temperature (22 °C) for 40 days (n = 1). During this time, we repeatedly sampled the gas from the syringe to analyze the CH_4_ concentration and its isotopic composition. The syringe was fitted with the same components as those used in the field experiment (a three-way stopcock, an injection port, and a hot melt adhesive sealing) to test the integrity of the entire assembly.
(2.3) To estimate the contribution of CH_4_ oxidation to the decrease of CH_4_ in trapped bubbles, we determined the specific fractionation factor (α) associated with CH_4_ oxidation in streamwater experimentally and applied it to the model of isotope fractionation developed for a closed nonsteady state system.^24^ Details of the experiment, related calculations, and the ^13^C-CH_4_ measurements are described in the Supporting Information.
(2.4) To estimate the effect of the redissolution of gas bubbles containing CH_4_ after they enter the bubble trap, we calculated the equilibration dynamics as described by Fick’s first law of diffusion:
dCgdt=−kAVgKH(Cg−Ceq)
  where *C*_g_ is the partial pressure of methane in bubble (atm), *t* is the time (days), *k* is the gas transfer coefficient for methane (m d^–1^), *A* is the surface area of the gas–water exchange interface (m^2^), *V*_g_ is the gas volume of accumulated gas in the bubble trap (mL), *K*_H_ is the temperature-dependent Henry’s solubility coefficient for methane (mol L^–1^ atm^–1^),^25^ and *C*_eq_ is the equilibrium partial pressure of methane (atm). The lower value of the gas transfer coefficient *k* (*k* = 0.1 m d^–1^) was used, which corresponds to the minimum effect of turbulence expected in our experimental setup.^26,27^

(3) For CH_4_ content comparison of naturally versus artificially released bubbles, we deployed six traps in the stream one month before actual sampling. On the days of sampling, all traps were completely refilled with streamwater. We monitored the traps using binoculars from the stream bank. Immediately after a bubble event spontaneously reached one of the traps, we collected a bubble sample from an inflatable boat. Simultaneously, artificially released bubbles were sampled by manually disturbing the sediments near the trap. Gas samples were measured for CH_4_ concentration in the field, and the remaining part of each sample was stored in 12 mL vials (Labco, U.K.) for later analysis of δ^13^C-CH_4_.

**Table 1:** Characteristics of the Stream Sites (Mean ± SD, If Relevant) during the Sampling Period

parameter/stream	CZ-Jihlava^a^	CZ-Hana^a^,^b^	CZ-Kyjovka^b^,^c^	AT-Schweinsbach^a^
GPS coordinates of the site	49.0261778N, 16.5187103E	49.2795372N, 17.0532986E	48.6824044N, 16.9372486E	48.1109167N, 15.3192222E
Strahler order	7	4	5	1
geology	granite/gneiss	clay	sandstone/claystone	clastic sedimentary material
trap deployment period^a^	25/6 to 25/7/2024	25/7 to 26/8/2024	na	16/10 to 15/11/2024
box experiment period^b^	na	29/7 to 20/8/2024	28/8 to 10/9/2024	na
stream width (m)	19.5	5.5	15	2
water depth (cm)	44.0 ± 11.8	57.2 ± 22.8	95.5 ± 47.2	42.7 ± 19.1
stream velocity (m s^–1^)	0.2 ± 0.1	0.03 ± 0.03	0.1 ± 0.04	0.1 ± 0.1
pH	7.6 ± 0.1	7.4 ± 0.3	7.3 ± 0.5	8.0 ± 0.1
conductivity (μS cm^–1^)	519.4 ± 31.6	880.9 ± 68.4	619.6 ± 5.0	641.8 ± 19.5
dissolved oxygen (mg L^–1^)	8.2 ± 0.5	7.3 ± 0.5	4.5 ± 0.4	10.0 ± 0.6
dissolved oxygen (%)	95.7 ± 3.8	80.2 ± 6.8	52.1 ± 5.9	89.8 ± 7.0
water temperature (°C)	22.4 ± 1.4	18.6 ± 1.0	21.4 ± 1.6	9.7 ± 2.4
dissolved CH_4_ (μg L^–1^)	8.4 ± 0.1	18.4 ± 0.5	20.2 ± 0.7	3.3 ± 0.1
catchment land use: forest/agriculture/urban/rest (%)	28/66/5/1	31/62/7/0	27/64/7/1	16/84/0/0

a Streams where the experiments for a number of the traps and length of the measurement period were conducted.

b Streams where the experiments on CH_4_ concentration changes within traps over time were conducted.

c Stream where the comparison of naturally and artificially released bubbles was conducted.

### Data Analyses

We simulated hypothetical sampling scenarios to determine the effect of the sampling period length and the number of bubble traps on the uncertainty of CH_4_ ebullition estimates. Based on this, we estimated the minimum number of bubble traps and sampling days required to achieve ±20% of the mean for a total number of traps or full sampling period. While the ±20% threshold is an arbitrary range of high accuracy in accordance with previous studies, it provides a reasonable balance between accuracy and uncertainty, given the spatial heterogeneity and temporal variability characteristic of ebullition measurements.^14,15^ For each stream, we generated data sets structured into bins. The number of bins corresponded to the maximum number of bubble traps (20) or sampling periods (10). All possible combinations of sampling period or trap ebullition means were generated and included in each bin (i.e., each possible number of bubble traps or sampling periods). The total number of unique combinations of a selected subset of *t* traps is defined by the binomial coefficient $\left(\begin{array}{l} N\\ t \end{array}\right)$ , where *N* represents the total number of available traps. In this study, the maximum number of possible combinations was $\left(\begin{array}{l} 20\\ 10 \end{array}\right)=184,756$ . Thus, the first bin in each set represented one trap and included all possible outcomes (i.e., all trap-specific ebullition means), and the last bin represented one possible outcome—the mean ebullition from the total number of traps. The bins in between each consisted of all *n* combinations of trap ebullition averages, with n increasing by (*n* + 1) per bin. This represents how the possible outcomes (the uncertainty in ebullition) converge toward the overall mean as the number of bubble traps increases. When all possible combinations reached the threshold of ±20% of the average ebullition, the number of bubble traps was assumed to be sufficient. The same approach was used for a temporal analysis to determine the optimal number of sampling periods. Sampling was conducted at 3–4-day intervals; therefore, individual bins represented sampling periods rather than individual days. Consequently, a total of ten bins were used to represent the full 30-day measurement period. This analysis was performed using MATLAB software. Additionally, we applied an asymptotic root-mean-square error (RMSE) model to extrapolate beyond the maximum number of traps used in our study and estimate the optimal number of traps, independent of sampling design limitations. This approach involves taking progressively larger subsamples (*n* = 1000 iterations per sample size) of the measured data for each stream to determine the optimal sampling effort. The RMSE model predicted absolute and relative errors in mean CH_4_ ebullition and identified the point at which increasing the number of traps resulted in diminishing returns (e.g., when the expected deviation from the mean fell below ±20%). This analysis was conducted in R^28^ using minpack.lm R package and the nlslm function.^29^

The differences in CH_4_ concentration and δ^13^C-CH_4_ between naturally and artificially released bubbles were tested using the Mann–Whitney rank sum test and Student’s *t*-test, respectively. The relationship between bubble volumes and their associated CH_4_ content was evaluated using Spearman’s correlation.

## Results and Discussion

### A Wide Variety of Sampling Designs for Lotic CH_4_ Ebullition Measurements across Past Studies

We identified 42 studies measuring CH_4_ ebullition from lotic aquatic ecosystems,^30^ mostly from the Northern Hemisphere, primarily in North America, Europe, and China (Figure 2a), reflecting a common geographic bias in inland water greenhouse gas research.^3^ Our extensive literature review showed that floating chambers and bubble traps were used almost equally, accounting for 45% and 55%, respectively (Figure 2b). Although the mean number of replicates used to estimate CH_4_ ebullition from individual studies is comparable for bubble traps (7.6 ± 7.0) and floating chambers (5.8 ± 7.5), deployment times differ considerably, with 101 ± 126 days for bubble traps and 102 ± 325 min for floating chambers (mean ± SD; Figure 3). The high variability across studies, as indicated by large standard deviations, underscores the lack of standardized sampling designs for measuring fluvial CH_4_ ebullition and highlights the urgent need for standardization. Additionally, trap and chamber sizes vary considerably, ranging from 0.008 to 0.79 m^2^, across a variety of environments. This indicates that the number of traps or chambers alone does not accurately reflect the spatial representativeness of measurements. The mean share of ebullition fluxes to total CH_4_ fluxes across all studies and methods used was 52 ± 30%, which nicely corresponds to the numbers found by Stanley et al.^3^ However, the share ranged from measuring zero fluxes up to 98%, suggesting that CH_4_ ebullition is a highly variable in streams and rivers.

**Figure 2:**
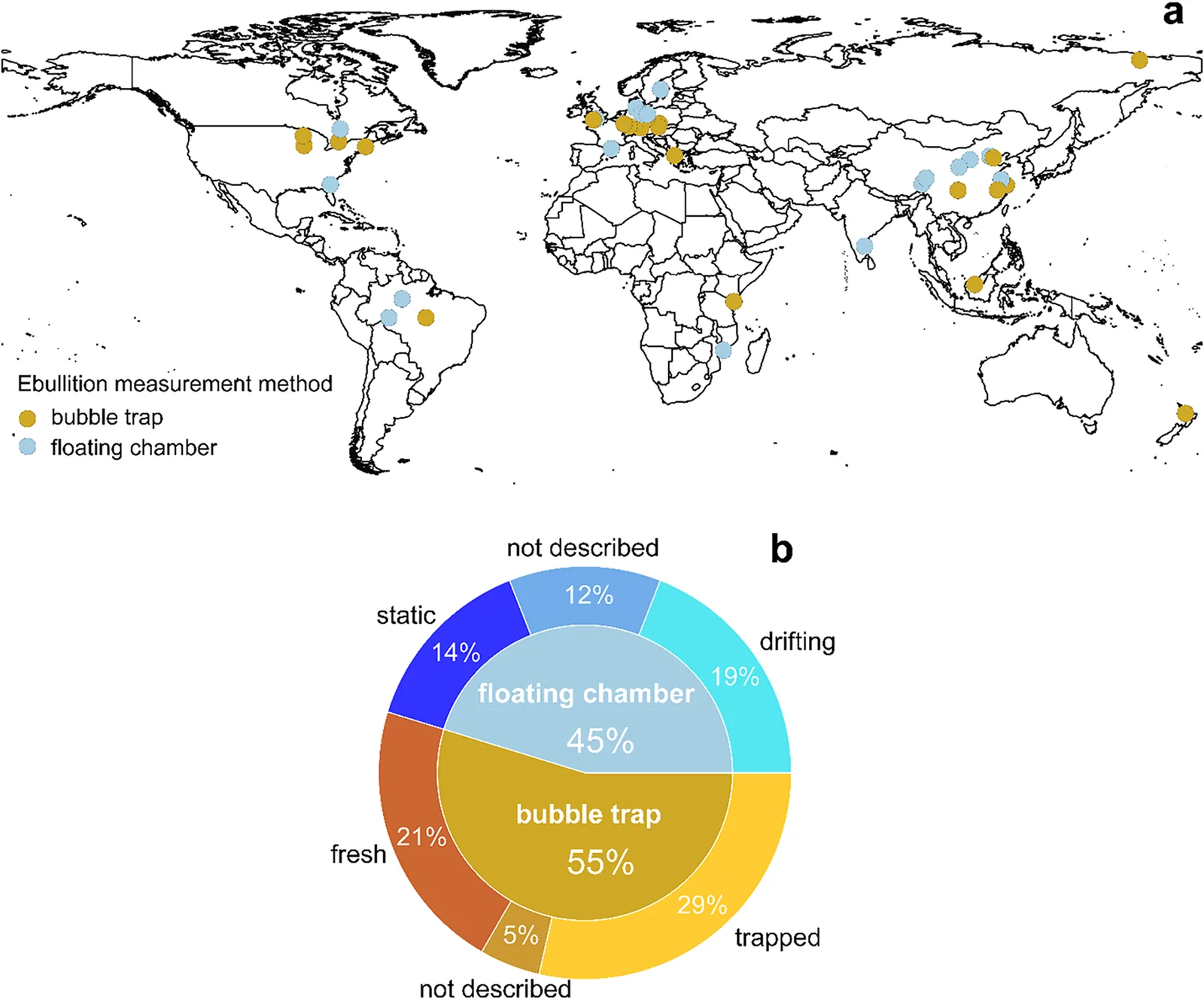
(a) Global distribution of study sites where fluvial CH_4_ ebullition was measured using bubble traps (brown) and floating chambers (blue) and (b) relative representation of ebullition methods across studies, differentiating between different types of floating chamber measurements (static versus drifting) and bubble CH_4_ content methods (fresh = artificially stirred from sediments versus trapped bubbles).

**Figure 3:**
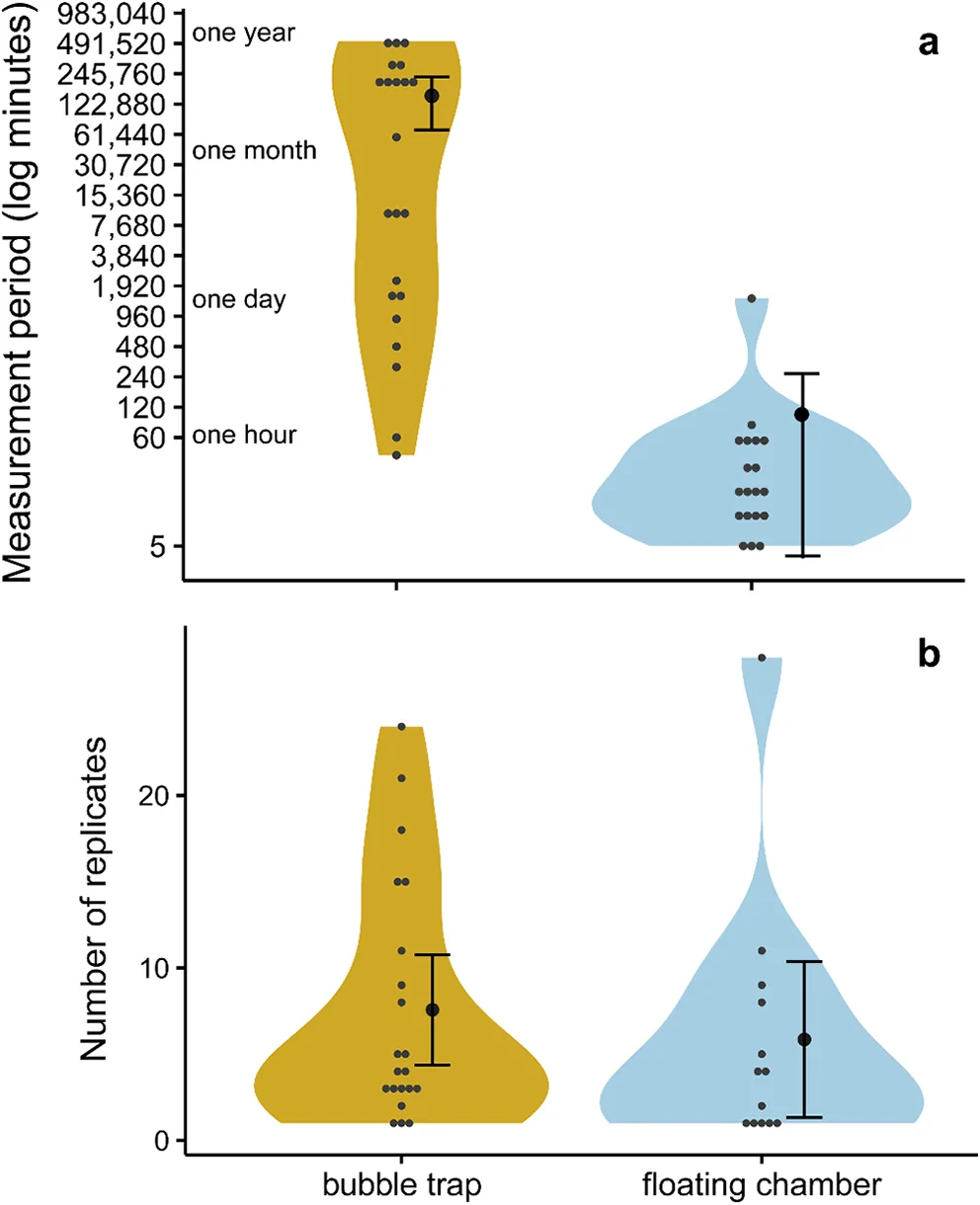
(a) Lengths of the deployment time and (b) the number of replicated bubble traps or floating chambers used to estimate fluvial CH_4_ ebullition across previous studies. The violin plots display Kernel density distributions, and the smaller black dots are the individual studies (differ between (a) and (b) as some studies did not report the number of replicate measurements, especially for floating chamber measurements). The bigger black dots and lines show the mean and standard deviation for each group.

### Accurate CH_4_ Ebullition Estimates in Highly Heterogeneous Streams Require Larger Number of Bubble Traps Than Typically Used

Simulations from three different streams indicate that 12–24 days of measurements with at least 19 bubble traps per stream achieve CH_4_ ebullition estimates within 20% deviation of the mean (Figure 4). Our results suggest that fewer traps cause greater deviation from the mean than shorter deployment times (Figure 4). Our minimum trap estimate (*n* = 19) across all three streams overlaps with the maximum number of traps used in our field measurements (*n* = 19–20). This suggests that our estimate may be constrained by the sampling design. Nevertheless, it exceeds estimates by Robison et al.^14^ for streams (*n* = 10 based on 12 traps) or Wik et al.^15^ for northern lakes (*n* = 11 based on 17 traps), despite the relatively narrow widths of our streams (2–20 m). Consistent with this, extrapolative validation of the asymptotic RMSE fit indicated that achieving a deviation of less than 20% from the mean CH_4_ ebullition would require substantially more traps than were deployed in this study (43, 73, and 206 traps for the Hana, Jihlava, and Schweinsbach, respectively). However, the asymptotic response curves suggest that most of the uncertainty reduction is achieved with approximately 15–20 traps in our investigated streams. Beyond this range, the curves approach an asymptote, and additional sampling effort yields only marginal reductions in measurement uncertainty (Figure S4). The substantially larger optimal trap estimate for Schweinsbach can likely be attributed to the highly skewed distribution of CH_4_ ebullition and its overall fluxes, which were 2 orders of magnitude lower than in the other streams. Most traps recorded very low or zero emissions, while total ebullition was dominated by two active spots.

**Figure 4:**
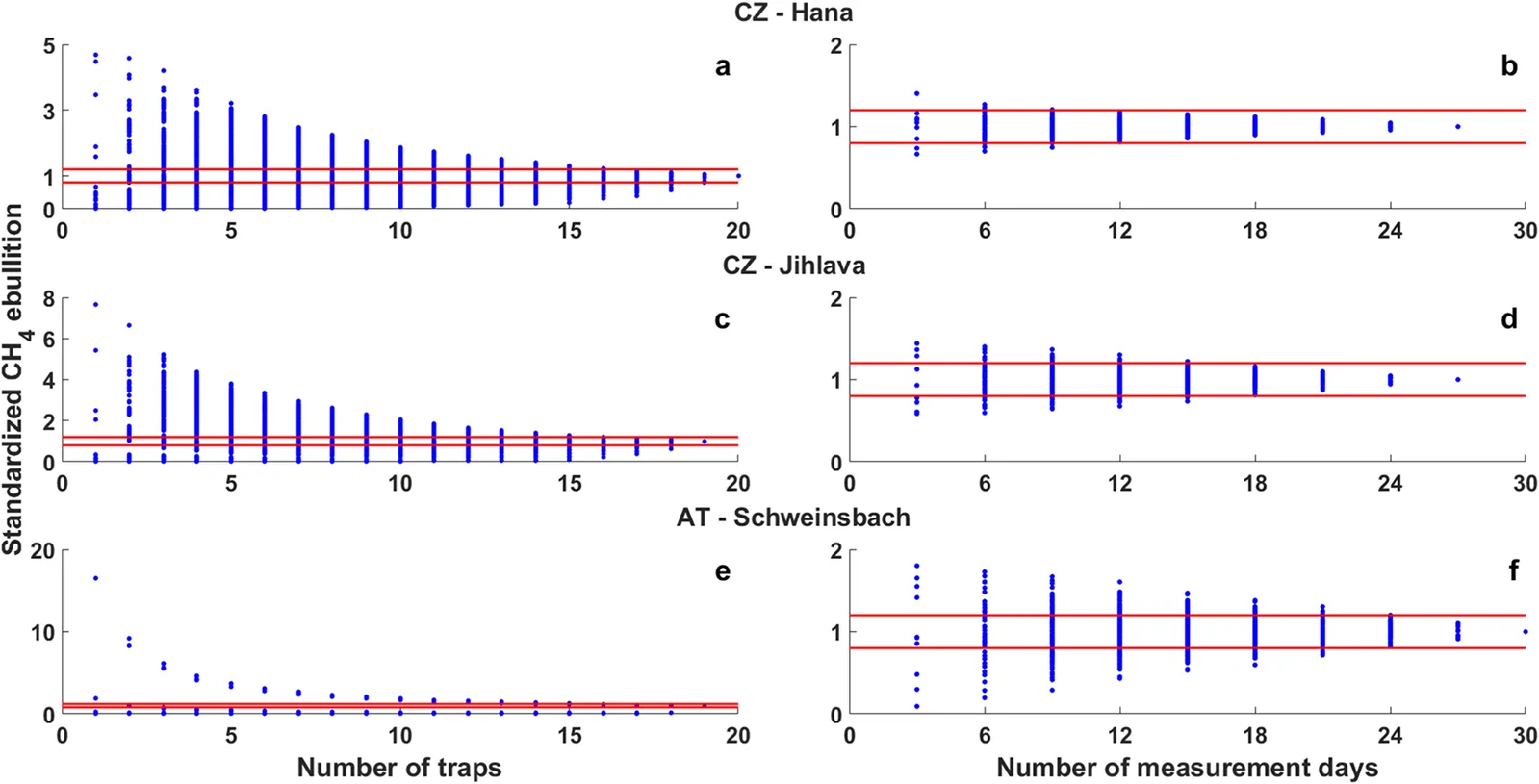
Simulation of different sampling regimes showing the range of deviation from the standardized CH_4_ ebullition mean for different numbers of traps (a, c, e) and different numbers of sampling days (b, d, f). Standardization was performed by dividing each individual measurement by the mean of the complete data set, resulting in a reference mean equal to 1. Red lines indicate 20% deviation from the standardized mean (mean = 1). Each pair of panels (i.e., a-b, c-d, e-f) represents a different stream. Analyses were performed by simulating all possible combinations of measurements. Note that the CH_4_ ebullition measurement of the last stream (e, f) was made one month after the flood event and at lower temperatures.

The large variability in CH_4_ ebullition likely stems from small-scale heterogeneities in the streams associated with the drivers of ebullition (e.g., sediment characteristics, temperature, water depth, or flow velocity).^14,31,32^ Sediment characteristics, including the quantity and quality of organic matter and the proportion of fine particles, primarily regulate CH_4_ production by controlling substrate availability and the probability of anoxic conditions.^33^ Temperature further drives mainly temporal variability in ebullition by stimulating microbial CH_4_ production in sediments and, to a lesser extent, by affecting gas solubility.^34^ Shallow water depths may promote ebullition through the accumulation of fine, organic-rich sediments in shallow stream reaches and through reduced hydrostatic pressure, with pressure fluctuations potentially triggering bubble release from sediments.^9,14^ Finally, low flow velocity can enhance the deposition of fine particles and organic matter while limiting oxygen supply to sediments, thereby further supporting methanogenic conditions.^35^ Therefore, the representativeness of ebullition estimates appears to depend more on the spatial heterogeneity of the examined ecosystem than on the specific number of traps.

Taking the surface area of the 100-m-long stream sections into account, our sampling design recommended a number of bubble traps that covered 0.1–0.8% of the total area examined. This corresponds well with the bubble traps’ spatial coverage of ∼0.4% (number derived from method description) recommended by Robison et al.^14^ for small streams. Meanwhile, the recommended number of bubble traps for lakes (surface area ranging from 1 to 17 ha) by Wik et al.^15^ corresponds to a considerably lower spatial coverage of 0.001–0.02% of the total lake area, which may reflect the expected lower small-scale spatial heterogeneity of lake sediments compared to streams. Nevertheless, the recommended number of traps characterizes CH_4_ ebullition in specific sections of the stream. Repeated measurements are required to ensure the representativeness of the ebullition estimates if environmental conditions vary in other stream sections.

In our study, many of the accumulated gas samples in bubble traps (*n* = 87) collected within 3 days contained <10% of CH_4_ (Figure S5). However, both the CH_4_ content of bubbles (0.01–67%) and the volume of captured bubbles (0–4.8 L m^–2^ d^–1^) were highly variable and likely contributed substantially to the observed variability in CH_4_ ebullition. There was no consistent relationship between bubble volume and CH_4_ content across streams, with a positive correlation at Hana and Jihlava streams (*r* = 0.66 and 0.46, *p* < 0.01) and no correlation at Schweinsbach stream (*p* > 0.4). Importantly, replacing trap-specific CH_4_ concentrations with the mean concentration measured at each site (*n* = 38–45) resulted in an underestimation of mean CH_4_ ebullition by 38–50% across the three streams. In contrast, using the mean CH_4_ concentration of the two freshly released bubble samples collected at each site as a representative value resulted in overestimation by 53–3458%, with the largest relative bias occurring at the site with very low CH_4_ ebullition (Schweinsbach). Because only two fresh-bubble samples were available per site, this scenario should be considered illustrative rather than a robust estimate of site-level bias. The wide range of CH_4_ content in the bubbles, as well as the high occurrence of very low CH_4_ contents, corresponds well with the previously reported range of values (0.01–98%), with the lower CH_4_ fractions mainly observed in sandy sediments rather than in muck.^5,21,31^ These results emphasize the importance of pairing measurements of bubble volume with corresponding CH_4_ content rather than relying on a single representative CH_4_ concentration.

The recommended optimal number of traps was consistent across all three streams, but the optimal sampling period length differed between streams. A recommended period of 12 or 18 days resulted from streams with minimal water temperature changes over a month of trap deployment (18–20 °C and 20–24 °C, respectively). A longer period of 24 days was recommended for Schweinsbach stream, where the first attempt at measurement was thwarted by a flood event, and the subsequent deployment one month after the flood had a water temperature range of 6–12 °C. We also could not exclude the effect of sediment redeposition after the flood. The considerably longer optimum periods of 60 and 39 days estimated by Robison et al.^14^ for streams and by Wik et al.^15^ for lakes, respectively, were based on sampling over 4 months, where temperature changes likely influenced the temporal variability of CH_4_ ebullition.^34^ In our case, minimal water temperature changes over the entire 30-day period suggest that other factors drove the temporal variability of ebullition. Short-term deployment of bubble traps is likely to miss short episodes that trigger the sudden release of accumulated bubbles, for example due to pressure changes or significant fluctuations in water depth.^16,36,37^ Therefore, the sampling period recommended based on our experiments applies to a specific season. Longer campaigns or repetition in other seasons are essential for annual ebullition estimates, especially in cases of significant temperature and water depth fluctuations between seasons.

### Bubble CH_4_ Loss from Traps Is Primarily Driven by Redissolution, Followed by Microbial Oxidation and Syringe Leakage

Between 3 and 7% of the initial bubble CH_4_ content was lost from traps per day, and δ^13^C-CH_4_ increased, along with the decreasing concentration over time (Figure 5). The CH_4_ concentration decrease corresponds to a first-order rate constant *k* ranging between 0.05 and 0.22 d^–1^. The lower bubble CH_4_ content and enriched δ^13^C-CH_4_ suggest redissolution of CH_4_ (gas exchange between oversaturated syringe headspace and underlying water) and the involvement of microbial oxidation in the removal of CH_4_ from the traps. The reduction in CH_4_ concentration due to redissolution alone was estimated using a theoretical calculation of diffusion, taking into account the size of the gas exchange interface and the volume of inserted bubbles. This led to a 2.5–4% decrease in the initial CH_4_ content per day, corresponding to a first-order rate constant of 0.04 d^–1^. On average, the redissolution accounted for 58 ± 12% of the observed CH_4_ loss from traps at the final phase of the experiment (Table S1). Nevertheless, the estimated contribution of redissolution is sensitive to the selected gas transfer coefficient k. Since we applied a low *k* value (*k* = 0.1 m d^–1^) corresponding to minimal turbulence conditions expected in our experimental setup, the calculated redissolution likely represents a conservative estimate.

**Figure 5:**
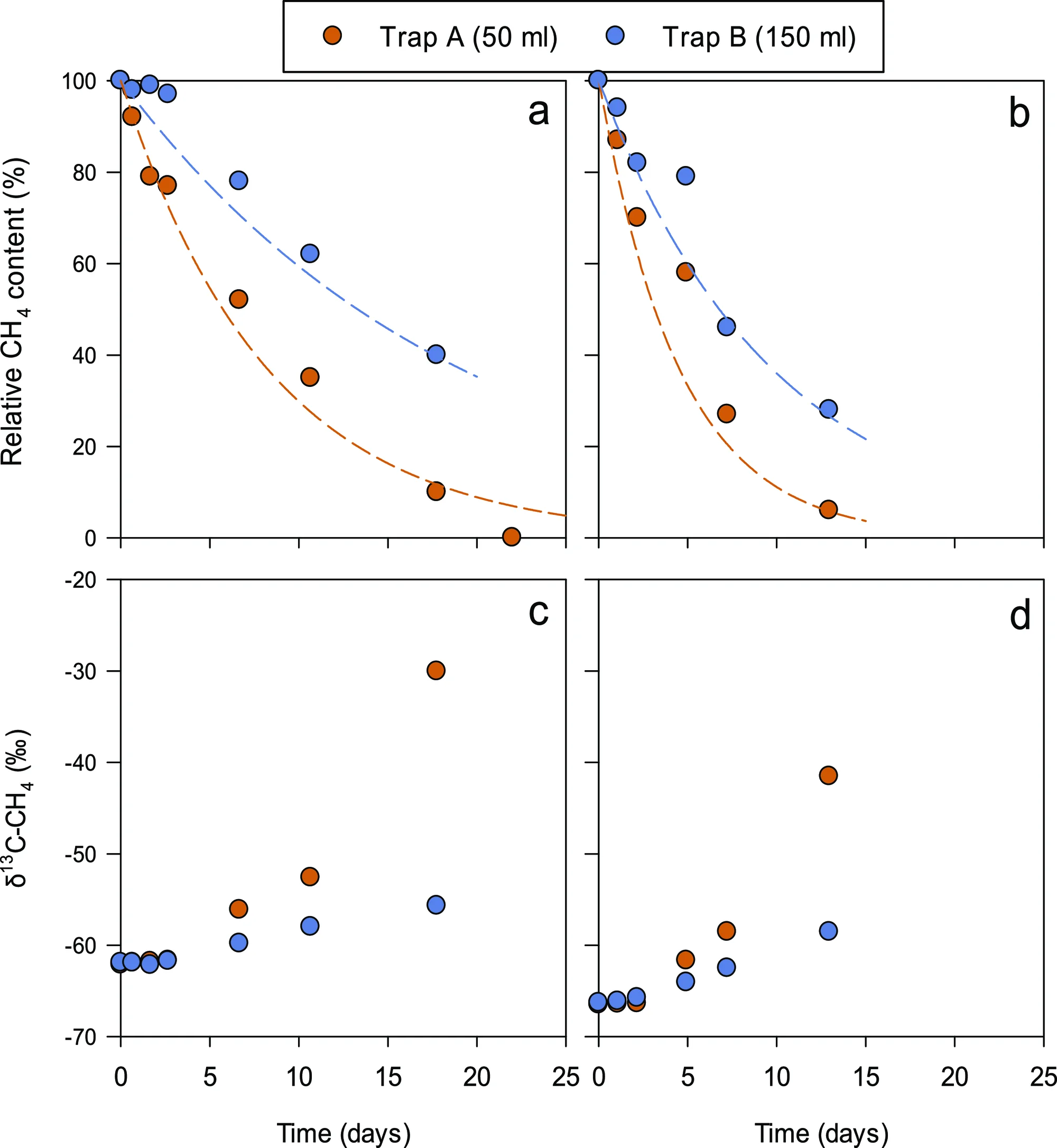
Relative CH_4_ content and its isotopic signature (δ^13^C-CH_4_) in bubble traps over time in the Haná stream (a, c) and the Kyjovka stream (b, d). The Kyjovka stream was characterized by a higher rate of CH_4_ oxidation in water compared to the Hana stream. Bubble traps were filled with 50 and 150 mL of gas containing ∼60% CH_4_. The dashed lines show the decrease in CH_4_ content that corresponds to the application of the first-order rate constant, which was derived from the observed decrease in CH_4_ concentration.

To estimate the effect of CH_4_ oxidation on the CH_4_ decrease in trapped bubbles, we determined the CH_4_ oxidation rate and the respective isotopic fractionation factor (αMOx) in both streams where the experiment was conducted. The CH_4_ oxidation rate in water was 0.24 μg L^–1^ h^–1^ with αMOx = 1.037 in the Hana stream and 1.8 μg L^–1^ h^–1^ with αMOx = 1.033 in the Kyjovka stream at in situ water temperatures of 19 and 21 °C, respectively. The experiment confirmed significant microbial CH_4_ oxidation in the water column, which also varied between streams. Furthermore, determining the site- and time-specific αMOx minimized the largest source of uncertainty when calculating the extent of CH_4_ oxidation based on isotope mass balance. On average, CH_4_ oxidation accounted for 33 ± 15% of the CH_4_ removal from the bubble traps at the final phase of the experiment, when the majority of CH_4_ had been removed. The estimated share of oxidation in overall CH_4_ removal was higher in the Kyjovka stream (59 and 30%) than in the Hana stream (27 and 17%). Since CH_4_ oxidation occurs at the air–water interface, the partial pressure of CH_4_ in the underlying water is reduced. This increases the concentration gradient between the syringe headspace and the water, which subsequently accelerates CH_4_ redissolution. In accordance with this, findings from lakes suggest that the estimate of CH_4_ oxidation based on the same stable isotope approach corresponds well with the difference in concentration between bubbles that have accumulated in traps over a certain time period and bubbles that have been stirred manually.^22^ On the other hand, contrasting results from a reservoir in which no change in CH_4_ content in accumulated bubbles was observed over 55 days^38^ suggest that environmental conditions or specific trap design features, such as the gas–water interface area, can affect the rate at which CH_4_ concentration changes in traps. Indeed, Kim et al.^39^ demonstrated that the original bubble gas composition and volume can be accurately modeled using the measured gas concentrations at the end of deployment, alongside other relevant parameters such as the surface area of the gas exchange interface, the gas-specific mass transfer coefficient, and the temperature-dependent Henry’s law solubility coefficient.

In addition, a minor part of the CH_4_ concentration decrease can be explained by the syringe leakage. The syringe leakage resulted in daily losses of 0.5% of the initial CH_4_ content (*R* ^2^ = 0.68, *p* < 0.05). Applying this decrease to our experimental data on changes in CH_4_ content in trapped bubbles over time led to syringe leakage accounting for an average of 14 ± 4% of total CH_4_ removal at the final phase of the experiment. Methane concentration decrease via leakage was associated with a shift in the δ^13^C-CH_4_ of 0.029‰ per day (*R* ^2^ = 0.91, *p* < 0.05; Figure S6). The isotopic fractionation α for the syringe leakage was 1.007. This light isotope fractionation is likely associated with the diffusion of CH_4_ through porous material, which is also associated with partial discrimination of the heavier stable carbon isotope ^13^C.^40^ A similar decrease in CH_4_ concentrations associated with a shift in the δ^13^C-CH_4_ was also reported during a sample storage test, when CH_4_ diffused through polytetrafluoroethylene (PTFE) and polypropylene (PP) plastic caps.^41^ Nevertheless, the actual importance of CH_4_ leakage may differ depending on the specific material used for the bubble accumulation part, as well as on the bubble trap setup (whether it is under or above the water). The most appropriate approach would therefore be to test the tightness of the specific setup before measuring to correct any potential issues.

In summary, we found that redissolution was responsible for the majority of the decrease in CH_4_ concentration in the trapped bubbles, followed by oxidation. Syringe leakage accounted for the smallest proportion of the decrease. These three processes together explained 94–115% of the actual decrease in CH_4_ concentration observed in the bubble traps in our experiment. In accordance with the theoretical assumptions of redissolution, the overall decrease in CH_4_ concentration was faster in traps with smaller bubble volumes (50 mL) than in traps with larger bubble volumes (150 mL) within both streams. Across both streams, the decline in both traps was faster in the stream with higher CH_4_ oxidation activity in the water (Kyjovka) than in the one with lower CH_4_ oxidation activity (Hana). This was consistent with estimates of variable CH_4_ loss due to oxidation based on the isotopic mass balance approach.

Other processes could further reduce CH_4_ concentration in bubbles, such as stripping, a process where less soluble gases (mainly nitrogen and oxygen) from the surrounding water can replace the more soluble gases initially present in the bubbles, which then dissolve into the surrounding water.^42,43^ To constrain the potential magnitude of this effect, we performed a scenario-based calculation of N_2_ and O_2_ transfer using the same gas–water exchange framework as for CH_4_ redissolution (Supporting Information: Estimation of CH_4_ dilution by N_2_ and O_2_ transfer into trapped bubbles). The measured dissolved O_2_ concentrations were used for each experiment, whereas dissolved N_2_ was assumed to range from 80 to 120% of atmospheric equilibrium saturation. Because the complete initial gas composition was not measured, we assumed that the non-CH_4_ fraction consisted predominantly of N_2_ and that the initial O_2_ content was negligible, consistent with the expected composition of bubbles originating from anoxic sediments.^44^

Across the considered scenarios, the influx of N_2_ and O_2_ resulted in a relative decrease of 0.7–1.3% of the initial CH_4_ content per day, corresponding to 11–30% of the observed CH_4_ decrease in bubble traps at the final phase of the experiment. This estimate represents dilution of CH_4_ through the addition of N_2_ and O_2_ rather than direct CH_4_ removal. Moreover, the influx of these gases occurs simultaneously with CH_4_ redissolution. These two effects are therefore coupled and cannot be directly summed or fully separated from the available data. Accordingly, the calculated range should be interpreted as a sensitivity estimate of the potential contribution of gas-induced dilution to the observed decline in CH_4_ concentration, rather than as an additional independent component of CH_4_ loss.

### Comparable CH_4_ Content and δ^13^C in Natural and Manually Stirred Bubbles

Mean CH_4_ concentration and δ^13^C-CH_4_ in naturally and artificially released bubbles were not significantly different, with 65.1 ± 13.0% and 64.8 ± 20.2% (*n* = 12), and −67.1 ± 1.3‰ and −67.4 ± 1.4‰ (*n* = 16), respectively (Figure 6) (*p* = 0.665 and 0.585, respectively). The CH_4_ content and δ^13^C-CH_4_ of naturally released bubbles and manually stirred bubbles from sediments have rarely been compared in the literature. We found a study by Martens and Chanton^45^ that also reported minimal differences in CH_4_ content of bubbles obtained by these two sampling techniques in a coastal lagoon. Similarly, the same authors compared bubbles sampled in a tidal freshwater estuary and observed no systematic difference in the isotopic composition between these two bubble types.^46^

**Figure 6:**
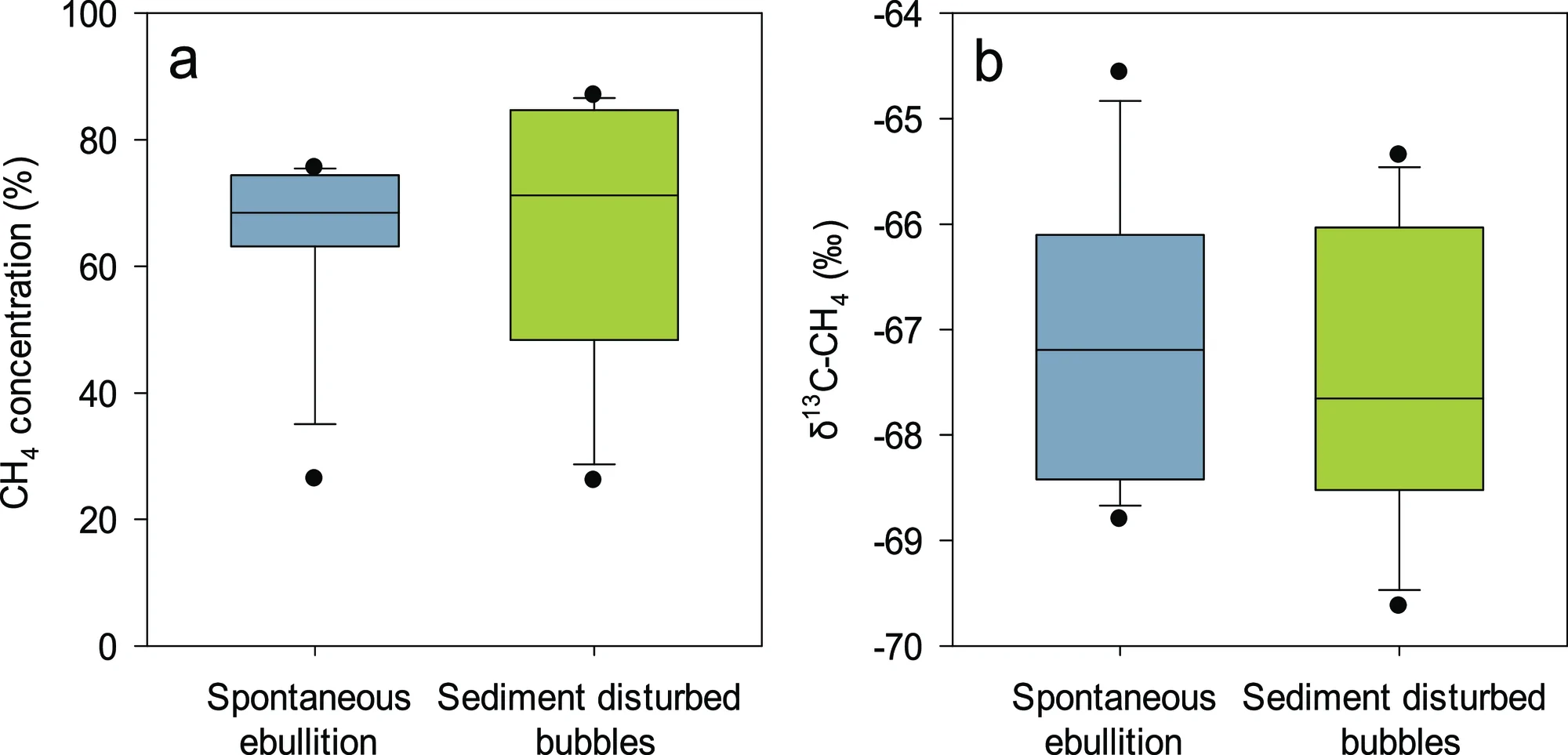
Comparison of CH_4_ concentration (a) and its isotopic composition (b) between spontaneously released bubbles by ebullition and artificially released bubbles by sediment disturbance in the Kyjovka stream. The sampling was carried out on three dates from May to August 2024. The boxes represent the median (horizontal line inside boxes), the first and third quartiles (upper and lower boundary of the box), the 10th and 90th percentiles (whiskers), and the outliers outside the range of the 10th and 90th percentiles (black dots).

### Uncertainty of Fluvial CH_4_ Ebullition Estimates

Our systematic review of previously adopted sampling designs in streams and rivers revealed high variability in the methods used and the setup of bubble traps in particular. In light of our results, the short deployment of floating chambers or the use of a very few bubble traps (<5), which we found across previous studies, might not adequately capture the high spatiotemporal variability of CH_4_ ebullition in streams. These findings also suggest that current estimates of fluvial CH_4_ emissions remain highly uncertain. The uncertainty of ebullition estimates increases substantially when the number of traps and deployment time are reduced. Based on our data, reducing the number of traps to 10 or 5 would result in average deviations from the reference CH_4_ ebullition estimate ranging from −98% to +94% and −100% to +259%, respectively (Figure 4). Applying the deviation estimated for seven traps to the mean sampling effort reported in our literature review (6.5 traps or chambers per site) suggests that recent CH_4_ ebullition estimates may, on average, deviate from the reference estimate by approximately −99% to +172% due to the limited number of bubble traps or floating chambers used. Similarly, the average deviation from the reference estimate increased as the measurement period shortened, from −9% to +10% for 24 days to −55% to +55% for 3 days, the shortest period evaluated in our study. Thus, the average deployment duration of bubble traps reported in our literature review (101 ± 102 days) appears sufficient to capture temporal variability. In contrast, the average deployment duration of floating chambers was only 102 ± 325 min, far below the shortest period evaluated here, suggesting that deviations due to limited temporal coverage could exceed the −55% to +55% range observed for three-day measurements. In addition, the use of a single representative CH_4_ concentration rather than paired measurements of bubble volume and CH_4_ content can introduce substantial bias in ebullition estimates, as demonstrated by our analysis above.

However, we do not attempt to derive a correction factor for ebullition estimates, unlike for diffusive fluxes where chamber-induced overestimation can be quantified from the relationship between near-surface turbulence and gas transfer velocity.^47,48^ The dominant source of uncertainty in ebullition measurements arises from the inherently episodic nature of ebullition, resulting in high spatial and temporal variability in the CH_4_ fluxes. Although trap design and sampling efforts can influence the resulting estimates, the magnitude and spatial and temporal patterns of ebullition are primarily controlled by environmental conditions and may differ substantially among habitats and ecosystems. Consequently, reduced sampling effort may lead to either over- or underestimation of ebullition fluxes rather than causing a systematic bias in one direction.

### Toward a Standardized Stream Ebullition Assessment for Accurate GHG Estimates

For the estimation of fluvial CH_4_ ebullition for a specific stream section and season, we recommend the deployment of at least 19 bubble traps over a period of 2–3 weeks. Even though we doubled the number of traps recommended previously, our recommendation still reached the maximum number of traps used in our study design. Therefore, a robust sampling design mainly needs to consider the large spatial heterogeneity of environmental parameters in streams (e.g., sediment characteristics, flow velocity, and water depth). Considering the high spatial variability of CH_4_ content in bubbles within the streams, we also strongly recommend trap-specific sampling of gas content conducted shortly after bubble accumulation (within 2–3 days). Our experiments clearly demonstrate that reducing the number of bubble traps results in a greater deviation in CH_4_ ebullition estimates than does reducing the deployment time.

While the optimal number of traps and deployment time may vary with environmental heterogeneity, other findings, such as the effect of CH_4_ depletion processes in traps and the similarity between naturally released and manually disturbed bubbles, can be applied when designing a sampling strategy in other ecosystems. In particular, there is a risk of a rapid decrease in CH_4_ concentration in the accumulated bubbles over time, primarily due to CH_4_ redissolution into the surrounding water and its oxidation, as suggested by results of our experiments and supported by previous studies documenting decreasing CH_4_ content in accumulated bubbles over prolonged sampling periods.^9,22^ Moreover, the composition of the gas in the bubble can be further affected by the equilibration process of other gases present in the surrounding water, such as nitrogen, oxygen, and carbon dioxide.^39^ These processes depend on the size of the trapped bubble and are more pronounced in small volumes. Therefore, if volumes of a few milliliters are anticipated, the sampling strategy should be adapted to ensure prompt sampling. At the same time, reducing the size of the gas–water exchange interface by modifying the trap design can minimize the effects of redissolution and oxidation. Sampling fresh bubbles by sediment disturbance circumvents this issue, but this method does not cover the high variability in CH_4_ contents observed between traps, resulting in potential over- or underestimation of CH_4_ ebullition. Nevertheless, this approach can be used as a complement, particularly for δ^13^C-CH_4_ analyses. Since ebullition is an episodic process, the entire bubble volume is not expected to be trapped during the entire period. Therefore, a reasonable estimate of the CH_4_ content in accumulated bubbles can be obtained after 2–3 days. At sites with a high probability of bubble accumulation within a short period of time, we recommend choosing a shorter gas sampling interval to minimize CH_4_ concentration changes.

Any correction to the original CH_4_ content is specific to the trap design and the environmental conditions. The main process responsible for the majority of the decrease in CH_4_ content in bubble traps is redissolution, which can be modeled based on theoretical calculations for specific bubble volumes and the size of the gas–water exchange interface. This is only challenged by the episodic nature of ebullition, when it is difficult to derive the original CH_4_ concentration in the trapped bubbles from the assumption of a linear decrease over time since the time of bubble release is unknown. However, Kim et al.^39^ present a modeling approach for deriving the original concentration of gas accumulated in bubble traps deployed in Canadian lakes, based on the accumulation time of bubbles in traps that occurs most frequently. The application of the same approach could be tested under highly heterogeneous conditions of small streams.

Moreover, high temporal resolution can be achieved by placing CH_4_ sensors in floating chambers or automated bubble traps with long deployment times.^7,10,49,50^ For long-term deployment of the floating chambers, the use of sensors to measure CH_4_ concentrations seems necessary because considerable CH_4_ oxidation and redissolution can be expected due to the large surface area of the gas exchange interface. The CH_4_ sensors solve the issue of changing CH_4_ concentrations in the headspace over time, but a sufficient number of chambers must be deployed across the study area to capture the considerable spatial variability of ebullition. Assuming adequate coverage of stream heterogeneity by automatic traps combined with sensors for accurate measurements of bubble volume and CH_4_ concentration appears to be a reliable approach, considering the CH_4_ content changes observed in our experiments. However, deploying automatic measuring systems may be more challenging in dynamic stream environments than in lentic waters because of water depth and flow turbulence constraints.

All parameters tested in our experiments, including the number of traps, deployment length, and the rate at which CH_4_ concentration changes in accumulated bubbles, may vary depending on the degree of heterogeneity of the environment under investigation and may be time- and site-specific. Regardless of the technique used, it is crucial to consider the large spatial and temporal variability of ebullition in heterogeneous streams as well as the possibility of rapid changes in CH_4_ concentration in traps. Consequently, the sampling strategy should be adapted to the characteristics of the systems being studied. For example, a larger number of traps may be required in natural streams and rivers with high sediment heterogeneity, where multiple interacting controls of ebullition create substantial spatial variability.^33,51^ In contrast, more homogeneous environments, often found in anthropogenically modified channels such as agricultural ditches or impounded river reaches upstream of weirs, may exhibit lower spatial variability and therefore require less intensive sampling effort.^52,53^ Here, we present a sampling framework based on field measurements under different environmental conditions. Following this systematic approach should improve the robustness and comparability of CH_4_ ebullition estimates among studies.

## Supplementary Material



## Data Availability

Data and metadata are available in the Zenodo data repository at 10.5281/zenodo.18743758 (Bednarik et al.^30^).

## Notes

Additional details on methods and results, including a systematic review of the sampling designs, analysis of gas samples, description of incubation experiments, estimated fraction of CH_4_ removal by individual processes during the bubble trap experiment, example of bubble traps deployment in streams, development of relative precision of CH_4_ ebullition estimates with increasing numbers of bubble traps, and CH_4_ content in the samples of gas accumulated in the bubble traps (PDF)
