# Supplementary material for: Chasing Bubbles: Toward a Standardized Approach for Quantifying Methane Ebullition Using Bubble Traps in Streams and Rivers
Source: Environ Sci Technol. 2026 Oct 23;60(44):31685–96. doi: 10.1021/acs.est.6c13075 (PMC13757941; doi:10.1021/acs.est.6c13075)
Supplement: Supplementary file 1 [file es6c13075_si_001.pdf]

Supplementary material for

Chasing bubbles: towards a standardized approach for quantifying methane ebullition using bubble traps in streams and rivers

Adam Bednařík[1], Eva Darenova[1], Helena Cvetkovic[2], Charlotte Doebke[2,3], Clemens Karwautz[2], Lukas Kokrda[1], Natalia Kowalska[1], & Katrin Attermeyer[2,3]*

[1] Department of Matters and Energy Fluxes, Global Change Research Institute of the Czech Academy of Sciences, Belidla 4a, 603 00 Brno, Czech Republic

[2] Department of Functional and Evolutionary Ecology, University of Vienna, Djerassiplatz 1, 1030 Vienna, Austria

[3] WasserCluster Lunz- Biologische Station, Dr. Carl Kupelwieser Promenade 5, 3293 Lunz am See, Austria

*Corresponding author: Adam Bednařík (bednarik.a@czechglobe.cz)

Contents of this file:

Number of Pages: 17

Number of text sections: 1

Number of Figures: 6

Number of Tables: 1

Text S1: Supplementary Methods

Details of systematic review of the sampling designs

We searched for papers measuring and reporting methane (CH_4) ebullition from running waters. The relevant papers were obtained from two different sources: a systematic review and private and online databases. We used the standardized "Preferred Reporting Items for Systematic Reviews and Meta-Analyses" (PRISMA) method to select studies to be included in the dataset (Page et al. 2021). The following inclusion criteria were defined: (i) to be published in English with (ii) original data and hence, reviews and other meta-analyses were excluded; (iii) to be performed in all types of streams and rivers, creeks or brooks and hence, reservoirs and standing water bodies as well as marine ecosystems were excluded; (iv) to include a method to measure or calculate methane ebullition and hence, papers only measuring diffusive or plant-mediated fluxes were excluded (Fig. S1). The search revealed papers in reservoirs, and we included these papers when they contained a fluvial site according to the site descriptions in the method sections of the papers (4 papers). The search also revealed three papers done in ditches, where CH_4 ebullition was measured, and we included these papers, too.

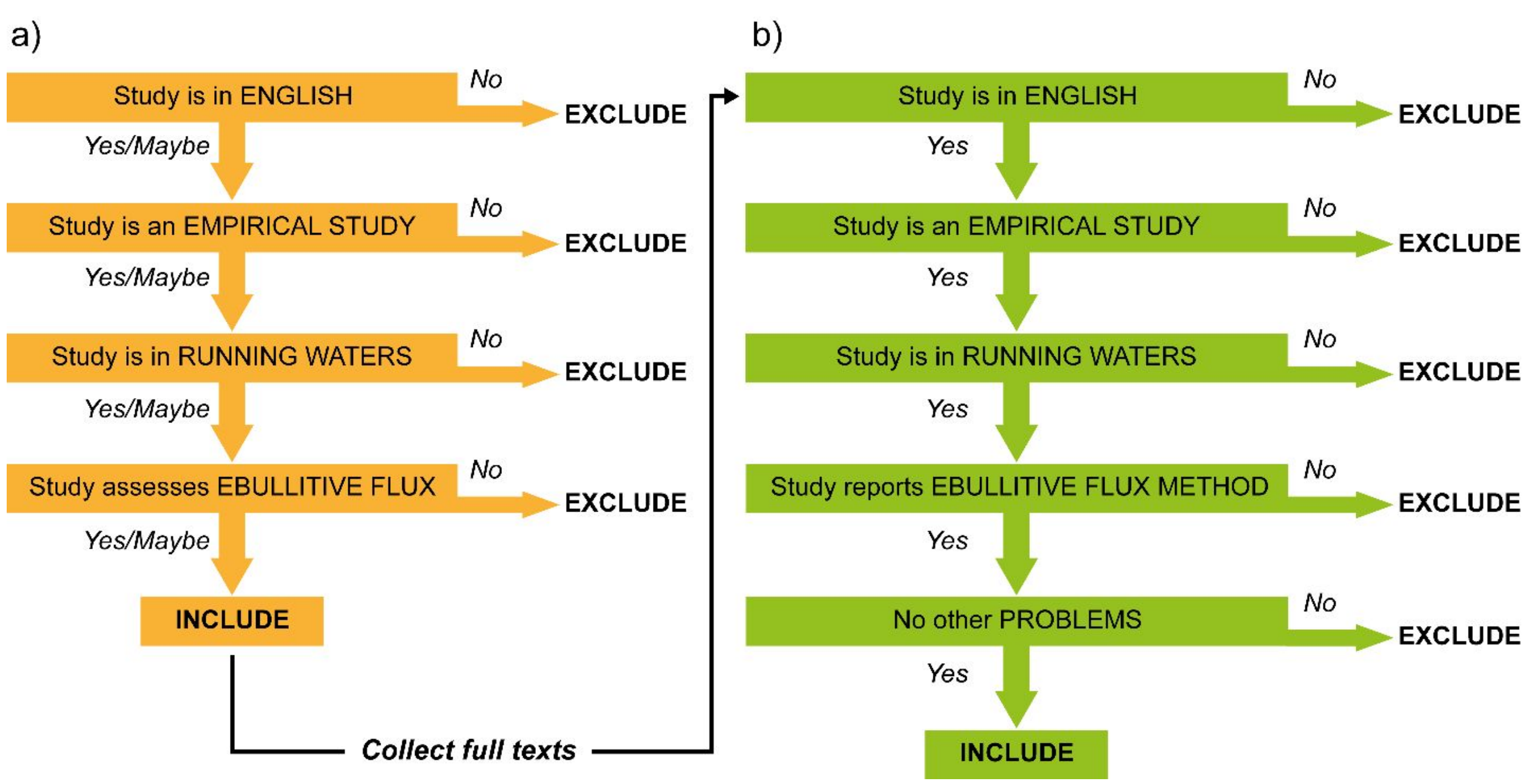

Figure S1. Initial title, abstract, and keywords (a) and full-text screening (b) decision trees for the list of studies assessing fluvial CH_4 ebullition.

For the systematic literature review, studies on fluvial CH_4 ebullition were identified through a comprehensive search of Web of Science and Scopus on November 29, 2024 using the following keywords limited to title and abstract: ((methan* OR ch4) AND (ebulli* OR bubbl* OR degas* OR outgas* OR flux* OR emission*) AND (stream* OR river* OR creek* OR brook* OR "flowing water*" OR "running water*" OR fluvial* OR lotic*)) and English (Languages); excluding subject areas Social, Economic, Medical or Computer Sciences). Keyword selection was based and refined on pilot screening and a comparison of studies collected by Adam Bednarik assessing CH_4 ebullition from streams.

The Web of Science and Scopus search identified 3,659 records after exclusion of 1.468 duplicate studies. Study titles and abstracts were skimmed for relevance, and we excluded 3,347 records that did not meet our inclusion criteria. Then, we excluded 271 records via full-text reviews based on the selection criteria previously described, leaving 41 unique studies for the dataset. From comparisons with the Global River Methane Database of concentrations and fluxes by Stanley et al. (2023) and our own collection, we added one more study that did not use any reference to running waters in their title or abstract (Herrero Ortega et al. 2019). From these studies, we extracted the description of the sampling method (including the number of traps or floating chamber incubations and the description of the method) as well as the share of CH_4 ebullition flux to total CH_4 flux directly from text, tables or by digitizing information from plots using WebPlotDigitizer software v.3.4 (Rohatgi 2020). For each study, we also extracted additional information related to geographic location, stream type and other descriptives such as stream order, width, watershed area, flow velocity and discharge, as well as sampling year and period (sampling year and mainly month within the sampling year). For the geographical coordinates, we provided site-specific values if available in the papers, but for studies conducted over large areas we provided an average latitude and longitude value. For the share of ebullition fluxes to total CH_4 fluxes, we averaged per study. In addition, we took the middle number if a range was given for the length of the deployment period and the replicate number of chambers or traps used to obtain one value for the figures. The full table is available in the Zenodo data repository at https://doi.org/10.5281/zenodo.15687421 (Bednarik et al. 2025).

Analysis of gas samples

We sampled the gas from bubble traps by withdrawing 15–20 ml of gas via the three-way stopcock at the top of the bubble trap using a 20 ml luer lock plastic syringe. Samples were injected into a 12 ml vial (Labco, UK) that had been previously flushed with zero air and pre-evacuated. The gas samples were analyzed for CH_4 concentration with a cavity ring-down spectroscopy (CRDS) gas concentration analyzer (Picarro GasScouter G4301, Picarro, CA, USA) and an ultraportable greenhouse gas analyzer (UGGA, Los Gatos Research, Inc., USA) using a closed-loop approach (Wilkinson et al. 2018). This method calculates the CH_4 concentration in the original sample based on the change in concentration after the sample injection into the closed loop created between the gas inlet and outlet of the analyzer. Depending on the expected concentration of CH_4, 0.01, 0.1, 0.5 or 1 ml of the sample was withdrawn from the vial using a gas-tight syringe (Hamilton, Switzerland) and injected into the closed-loop system. The CH_4 concentration was then calculated as follows:

$$CH_{4\_sam} = \frac{V_{eff} \times \Delta CH_4 + V_{sam} \times CH_{4\_post}}{V_{sam}} \quad (1)$$

where CH_{4_sam} represents CH_4 concentration in the sample, ΔCH_4 is the difference between the background CH_4 concentration in the loop before the injection and the equilibrium CH_4 concentration after the sample injection, V_{sam} is the volume of the injected into the loop, CH_{4_post} is the equilibrium CH_4 concentration in the loop after the sample injection, and V_{eff} is the effective volume of the closed loop.

The effective volume of the closed loop (V_{eff}) was estimated by injecting a calibration gas (Messer, Germany) with a known CH_4 concentration of 102.8, 1,001, 17,600 ppm or 99.99%, depending on the sample concentration and volume, in several replicates alongside each set of samples, and calculated as follows:

$$V_{eff} = \frac{V_{cal} \times (CH_{4\_cal} - CH_{4\_post})}{\Delta CH_4} \quad (2)$$

where V_{cal} is the volume of calibration gas injected into the loop, CH_4_cal is known CH_4 concentration of calibration gas, CH_4_post is the equilibrium CH_4 concentration in the loop after the sample injection, and ΔCH_4 is the difference between the background CH_4 concentration in the loop before the injection and the equilibrium CH_4 concentration after the sample injection.

Subsamples for stable isotopic composition of CH_4 ($\delta^{13}C$-CH_4) analysis were taken from the same vials that were previously measured for concentrations as described above. Gas samples were analyzed in the laboratory using a cavity ring-down spectroscopy (CRDS) analyzer (G2201-i, Picarro Inc, USA) equipped with a Small sample introduction module (SSIM2, Picarro Inc, USA) to determine $\delta^{13}C$-CH_4. Samples with high CH_4 concentration were diluted using hydrocarbon-free ultrapure zero air (Messer Technogas, CZ) to stay within the measurement guaranteed range of the analyzer and close to the concentration of standards used. The calibration of $\delta^{13}C$-CH_4 was performed using certified isotopic standards ($\delta^{13}C$-CH_4 = −68.6 ± 0.3‰, −45.5 ± 0.3‰, and −25.8 ± 0.3‰; Airgas, USA) and the measurements were corrected for instrumental drift during the measurement time using the same standards. The CH_4 isotopic data are reported in the standard delta notation (δ) expressed in ‰ relative to the Vienna Pee Dee Belemnite standard.

Description of an incubation experiment to determine the fractionation factor (α) associated with CH_4 oxidation in stream water

At two streams (CZ-Kyjovka, CZ-Hana), a set of eight 50 mL glass vials was filled with surface water (sampled 10 cm below the water surface), sealed with a butyl stopper and crimped with an aluminum cap. Two vials were immediately treated with 200 µL of 25% H_2SO_4 injected through the butyl stopper to prevent further microbial activity. These two vials corresponded to the initial surface water CH_4 concentration at the start of the incubation. The remaining vials were incubated in situ and the incubation was stopped by adding 200 µL of 25% H_2SO_4 to duplicate vials at three time intervals in the range of 0-24 h after the start of the incubation. Water samples were analyzed for dissolved CH_4 concentration using the headspace equilibration method, and subsamples of the headspace were stored in 12 ml vials (Labco, UK) for later analysis of $\delta^{13}C\text{-}CH_4$.

The α values were calculated using a simplified Rayleigh fractionation model for a closed system (Mariotti et al. 1981):

$$ln\left(\frac{CH_4}{CH_{4\_initial}}\right) = \frac{\alpha}{1-\alpha}\ln\left(\frac{1000+\delta^{13}C-CH_4}{1000+\delta^{13}C-CH_{4\_initial}}\right) \quad (3)$$

where CH_4 and $\delta^{13}C\text{-}CH_4$ are the concentration and isotopic signature of the CH_4 in the headspace at subsequent time points, and CH_4_initial and $\delta^{13}C\text{-}CH_4$_initial are the concentration and isotopic signature of the CH_4 at the beginning of the incubation.

The α value was derived using a fractionation model for a closed system, given that the experiment was conducted in crimped vials, preventing any exchange of substances with the surrounding water. The α was obtained by plotting Eq. (3) with $\ln(1000 + \delta^{13}C\text{-}CH_4)$ on the X axis and $\ln(CH_4)$ on the Y axis for a series of time points and performing a linear regression with a slope of $\alpha/(1-\alpha)$ as described by Mahieu et al. (2006).

The relative extent of CH_4 oxidation in bubble traps was estimated by a model of isotope fractionation developed for a closed steady state systems (Liptay et al. 1998): :

$$\ln(1 - f_{ox}) = \left[ln\left(\delta^{13}C{-}CH_{4\_source}{+}1000\right){-}ln(\delta^{13}C{-}CH_4{+}1000)\right]/(\alpha{-}1) \quad (4)$$

where fox is the fraction of CH_4 oxidized, $\delta^{13}C$-CH_4 is a subsequent isotopic signature of CH_4 in traps at different times of experiment, $\delta^{13}C$-CH_4_source is an isotopic signature of original CH_4, which was injected into the traps, α is the isotopic fractionation factor for CH_4 oxidation (eq. 3).

Estimation of CH_4 dilution by N_2 and O_2 transfer into trapped bubbles

The potential dilution of CH_4 in accumulated bubbles due to the transfer of dissolved N_2 and O_2 from the surrounding water was estimated using the same gas–water exchange framework as applied to CH_4 redissolution (Section 2.4). Gas-specific transfer coefficients for N_2 and O_2 were derived from the CH_4 transfer coefficient ($k_{L,CH4}$ = 0.1 m d^{-1}) using Schmidt-number scaling (Raymond et al. 2012):

$$k_{L,i} = k_{L,\,CH4}\left(\frac{Sc_i}{Sc_{CH4}}\right)^{-2/3} \quad (5)$$

where Sc_i is the temperature-dependent Schmidt number of gas i. Schmidt numbers for CH_4, N_2, and O_2 were calculated from the temperature-dependent polynomial relationships provided in Raymond et al. (2012). The exponent −2/3 was selected to represent gas transfer across a relatively smooth interface under the low-turbulence conditions of our experiment (Jähne et al. 1987).

For O_2, the measured dissolved concentrations in the water were used (7.3 mg l^{-1} and 4.5 mg l^{-1} for Hana and Kyjovka stream, respectively). Dissolved N_2 concentrations were not measured and were therefore calculated assuming 80, 100, and 120% of atmospheric equilibrium saturation, representing a range of plausible N_2 concentrations. Temperature-dependent Henry's solubility coefficients for N_2 and O_2 were obtained from Sander (2015). Because the complete initial gas composition of bubbles was not measured,

the non-CH_4 fraction of the initial bubbles was assumed to consist predominantly of N_2, with negligible initial O_2, consistent with the expected composition of bubbles originating from anoxic sediments (Langenegger et al. 2019). The transfer of N_2 and O_2 was calculated separately for each experimental bubble volume (50 and 150 ml), accounting for the corresponding gas–water interface area (5.7 and 14.5 cm^2), water temperature (19 and 21 °C), experimental duration (13 and 18 days), and dissolved gas concentration. To estimate the dilution effect independently of direct CH_4 removal, the initial amount of CH_4 was held constant. The resulting CH_4 mole fraction after N_2 and O_2 transfer was calculated as:

$$x_{CH4,t} = \frac{n_{CH4,0}}{n_{N2,t}+n_{O2,t}+n_{CH4,0}} \tag{6}$$

where $x_{CH4,t}$ is the CH_4 mole fraction in the bubble after time t, $n_{CH4,0}$ is the initial amount of CH_4 in the bubble, and $n_{N2,t}$ and $n_{O2,t}$ are the calculated amounts of N_2 and O_2 in the bubble after time t, respectively.

This calculation represents a sensitivity analysis of the potential dilution of CH_4 by N_2 and O_2 influx rather than an independent estimate of CH_4 removal. In the experiment, transfer of these gases occurred simultaneously with CH_4 redissolution, and the two effects therefore cannot be fully separated or directly summed.

Table S1. Estimated fraction of CH_4 removal by individual processes during the bubble trap experiment. The last column shows the actual CH_4 removal based on the decrease in measured CH_4 concentration (1 = 100%).

**Site/Bubble volume (ml)**	**Time (h)**	**Redissolution**	**Oxidation**	**Leakage**	**Redissolution + Oxidation + Leakage**	**Measured CH_4 loss**
CZ - Hana 50 ml	16	0.03	0.00	0.00	0.03	0.08
	40	0.07	0.01	0.01	0.09	0.18
	64	0.11	0.01	0.02	0.13	0.28
	160	0.24	0.01	0.05	0.30	0.55
	256	0.36	0.16	0.07	0.59	0.72
	**426**	**0.52**	**0.24**	**0.12**	**0.88**	**0.88**
CZ - Hana 150 ml	16	0.02	0.00	0.005	0.03	0.03
	40	0.06	0.00	0.01	0.07	0.08
	64	0.09	0.00	0.02	0.11	0.13
	160	0.21	0.005	0.05	0.26	0.29
	256	0.31	0.06	0.07	0.45	0.43
	**426**	**0.47**	**0.11**	**0.12**	**0.69**	**0.60**
CZ - Kyjovka 50 ml	26	0.04	0.003	0.01	0.06	0.21
	53	0.09	0.005	0.02	0.11	0.38
	119	0.19	0.15	0.03	0.37	0.66
	174	0.27	0.23	0.05	0.55	0.80
	**311**	**0.43**	**0.55**	**0.09**	**1.07**	**0.94**
CZ - Kyjovka 150 ml	26	0.04	0.005	0.01	0.05	0.10
	53	0.08	0.02	0.02	0.11	0.20
	119	0.16	0.07	0.03	0.27	0.40
	174	0.23	0.12	0.05	0.40	0.52
	**311**	**0.38**	**0.22**	**0.09**	**0.69**	**0.73**

* During the experiment, the proportion of CH_4 removal explained by the monitored processes increased, reaching 94–115% of the measured CH_4 decrease in the final phase: compare the fraction removed by all three processes (Redissolution + Oxidation + Leakage) and the measured CH_4 loss. The lower proportion of CH_4 removal explained by these three processes in the initial phases is probably due to small absolute changes in CH_4 concentration and its isotopic composition, which led to uncertainty in estimating the relative proportions of oxidation and dissolution. In addition, a scenario-based calculation indicated that the influx of N_2 and O_2 from the surrounding water could potentially reduce the relative CH_4

concentration through dilution. This effect is not included as an additional component of CH_4 removal because it does not directly remove CH_4 and occurs simultaneously with CH_4 redissolution; consequently, the contributions of these processes to the observed decrease in CH_4 concentration cannot be fully separated.

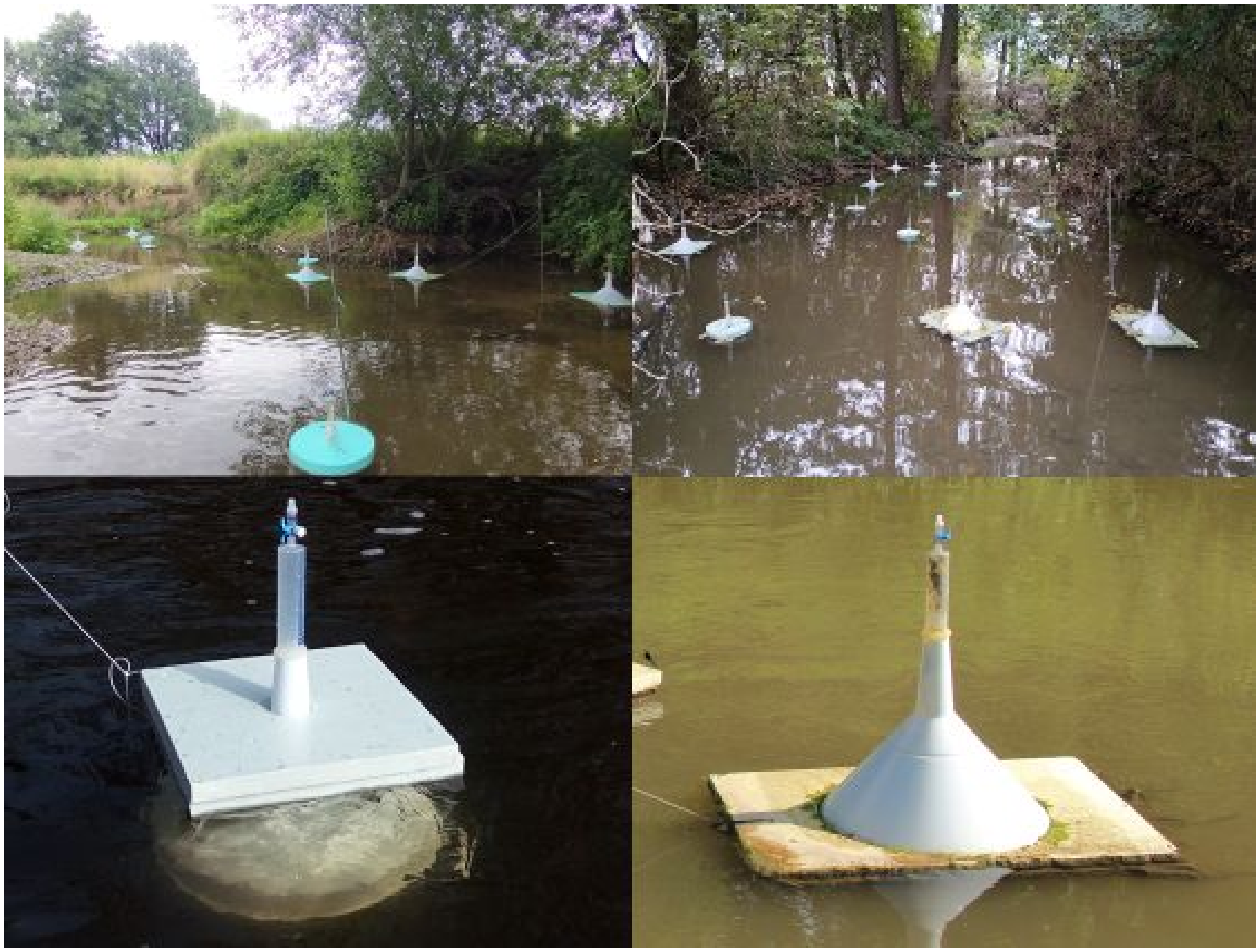

Figure S2. Example of bubble traps deployment covering a range of water depths and velocities in the Schweinsbach (top left) and Hana (top right) streams. To measure CH_4 ebullitive fluxes, a bubble trap consisting of an inverted plastic funnel (diameter: 33.8 cm; surface area: 0.09 m^2), floating material (Styrofoam) and a 60 ml plastic Luer-lock syringe with a three-way stopcock was used (bottom left). Another bubble trap design, using the same materials, was employed for deployment in very shallow zones (bottom right). The bubble trap was submerged by only a few centimetres (2–3 cm) to avoid disturbing the sediment.

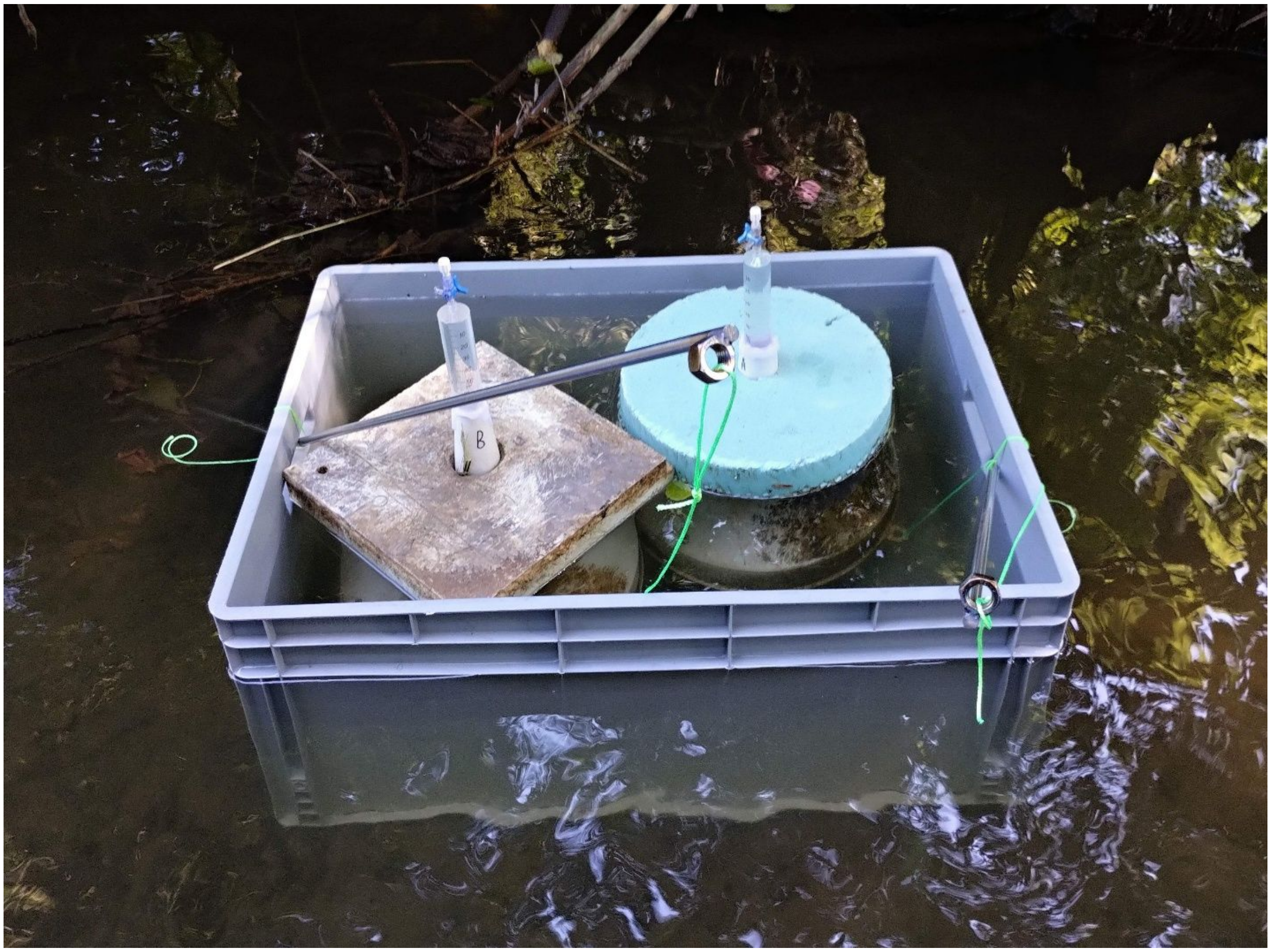

Figure S3. Picture of a box with a full bottom in a stream, where two bubble traps were placed to determine any potential changes in CH_4 concentration and its isotopic composition in trapped bubbles over time. The box was immersed in the stream water to keep the environmental conditions in situ (e.g. water and air temperature, pressure or microbial CH_4 oxidation). Water could exchange through two holes (50 cm^2) in the box, but naturally released bubbles could not reach the traps due to the full bottom of the box. The experiment was carried out in two streams (CZ-Hana, CZ-Kyjovka).

Supplementary Results

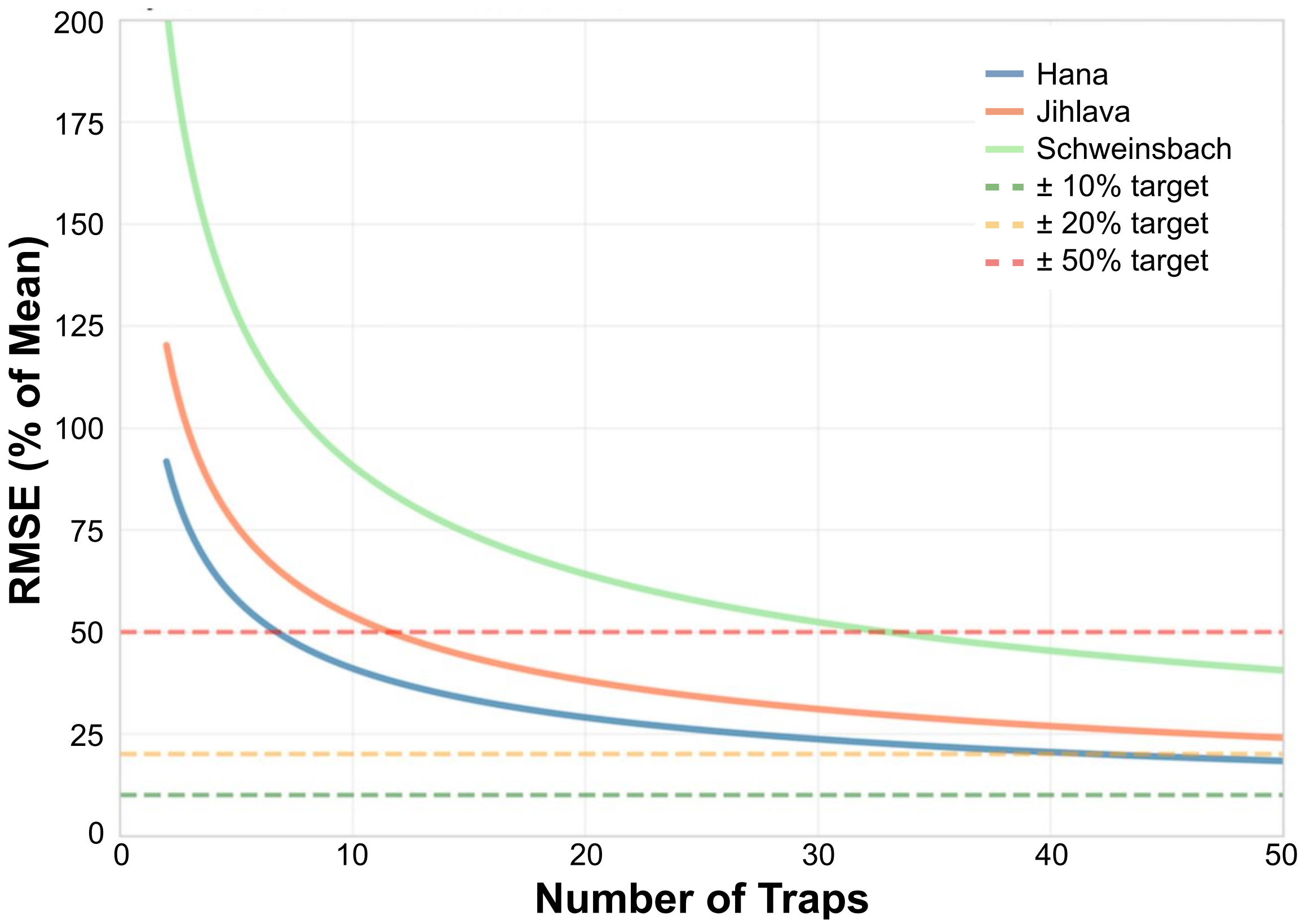

Figure S4. Development of relative precision (% of the mean) of CH_4 ebullition estimates with increasing numbers of bubble traps based on an asymptotic root mean square error (RMSE) model. Solid lines represent the asymptotic fits for individual streams. Dashed horizontal lines indicate target precision levels ranging from 10 to 50% of the mean. The intersection between the fitted curves and the 20% target precision line corresponds to 43, 73, and 206 traps for the Hana, Jihlava, and Schweinsbach, respectively.

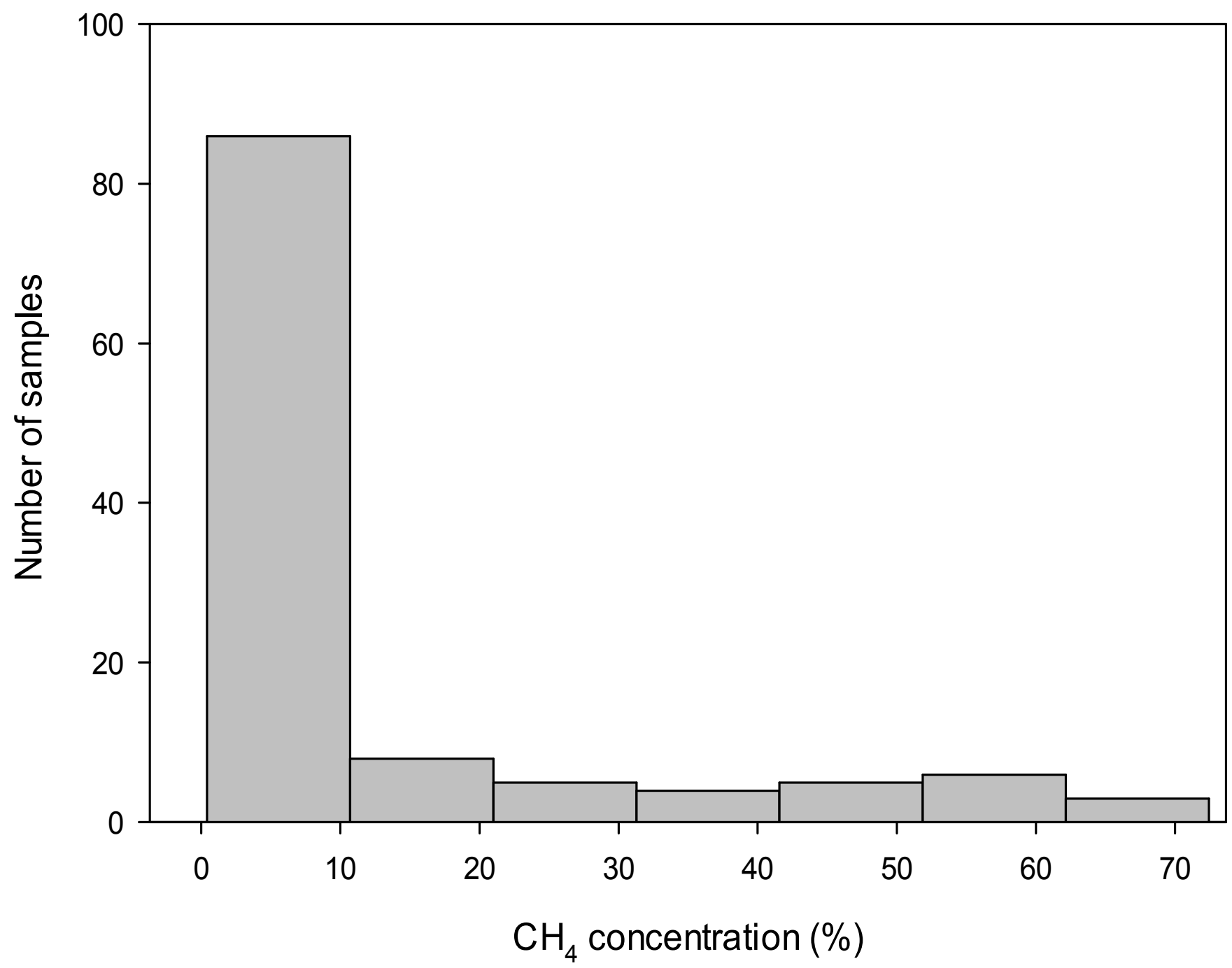

Figure S5. Histogram of the content of CH_4 in the samples of gas accumulated in the bubble traps (n = 117).

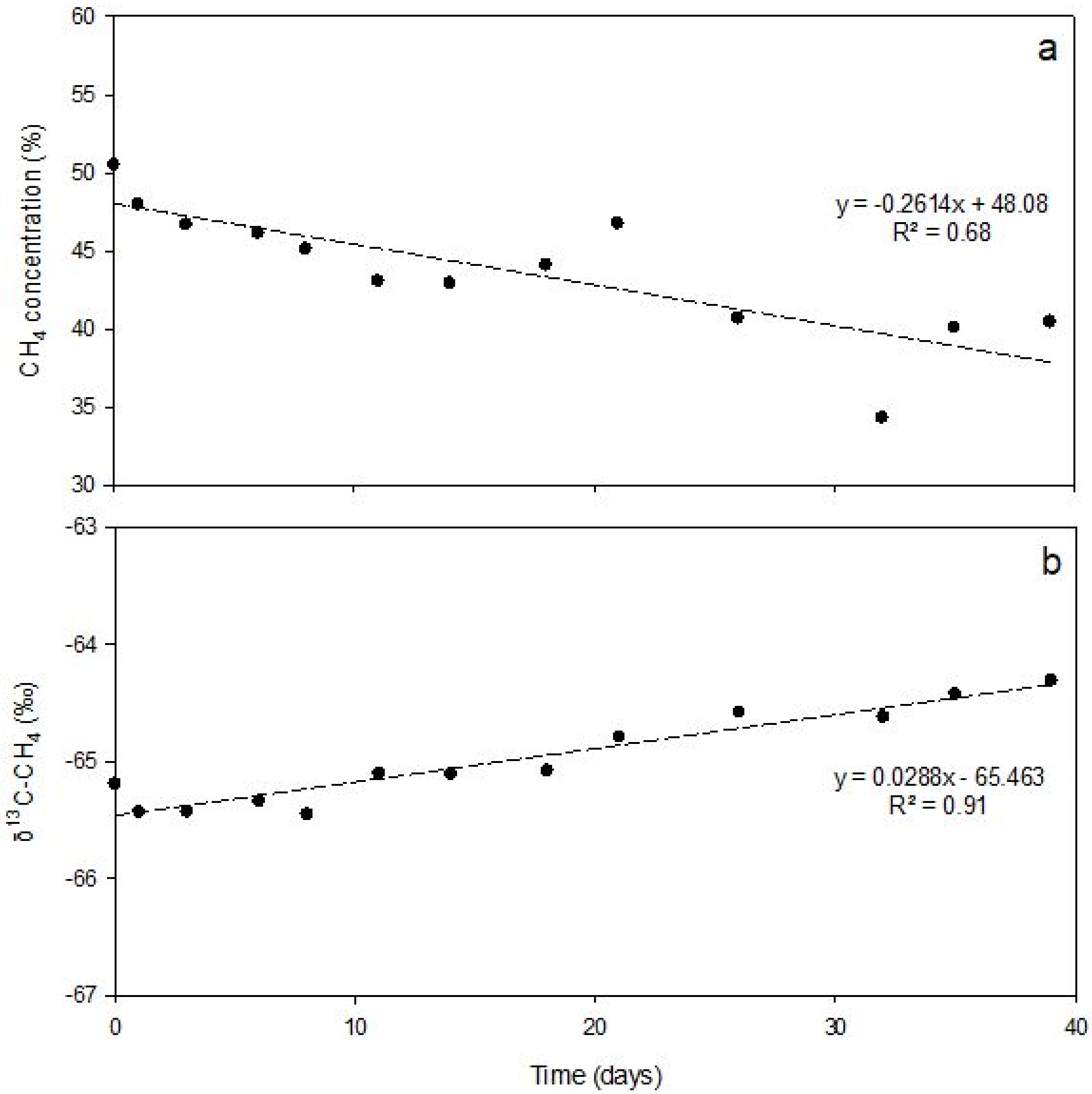

Figure S6. Changes in the concentration of CH_4 (a) and in its isotopic composition (b) in the closed syringe that was left at room temperature for 40 days. We used the same type of syringe that was used to make the bubble traps, as well as the glue, three-way valve and injection port.

Supplementary References

Bednařík, A., E. Darenova, H. Cvetkovic, C. Doebke, C. Karwautz, N. Kowalska, L. Kokrda, and K. Attermeyer. 2026. Chasing bubbles: towards a standardized approach for quantifying methane ebullition in streams and rivers [Data set]. Zenodo. https://doi.org/10.5281/zenodo.18743758

Herrero Ortega, S., C. Romero González-Quijano, P. Casper, G. A. Singer, and M. O. Gessner. 2019. Methane emissions from contrasting urban freshwaters: Rates, drivers, and a whole-city footprint. Glob. Change Biol. **25**: 4234–4243. doi:10.1111/gcb.14799

Jähne, B., K. O. Münnich, R. Bösinger, A. Dutzi, W. Huber, and P. Libner. 1987. On the parameters influencing air-water gas exchange. J. Geophys. Res. Oceans **92**: 1937–1949. doi:10.1029/JC092iC02p01937

Liptay, K., J. Chanton, P. Czepiel, and B. Mosher. 1998. Use of stable isotopes to determine methane oxidation in landfill cover soils. J. Geophys. Res. Atmos. **103**: 8243–8250. doi:10.1029/97JD02630

Mahieu, K., A. D. Visscher, P. A. Vanrolleghem, and O. V. Cleemput. 2006. Carbon and hydrogen isotope fractionation by microbial methane oxidation: Improved determination. Waste Manag. **26**: 389–398. doi:10.1016/j.wasman.2005.11.006

Mariotti, A., J. C. Germon, P. Hubert, P. Kaiser, R. Letolle, A. Tardieux, and P. Tardieux. 1981. Experimental determination of nitrogen kinetic isotope fractionation: Some principles; illustration for the denitrification and nitrification processes. Plant Soil **62**: 413–430. doi:10.1007/BF02374138

Page, M. J. and others. 2021. The PRISMA 2020 statement: an updated guideline for reporting systematic reviews. BMJ n71. doi:10.1136/bmj.n71

Raymond, P. A. and others. 2012. Scaling the gas transfer velocity and hydraulic geometry in streams and small rivers: Gas transfer velocity and hydraulic geometry. Limnol. Oceanogr. Fluids Environ. **2**: 41–53. doi:10.1215/21573689-1597669

Sander, R. 2015. Compilation of Henry's law constants (version 4.0) for water as solvent. Atmos. Chem. Phys. **15**: 4399–4981. doi:10.5194/acp-15-4399-2015

Stanley, E. H., L. C. Loken, N. J. Casson, S. K. Oliver, R. A. Sponseller, M. B. Wallin, L. Zhang, and G. Rocher-Ros. 2023. GRiMeDB: the Global River Methane Database of concentrations and fluxes. Earth Syst. Sci. Data **15**: 2879–2926. doi:10.5194/essd-15-2879-2023

Wilkinson, J., C. Bors, F. Burgis, A. Lorke, and P. Bodmer. 2018. Measuring CO2 and CH4 with a portable gas analyzer: Closed-loop operation, optimization and assessment V. Gupta [ed.]. PLOS ONE **13**: e0193973. doi:10.1371/journal.pone.0193973
